# Inhalation of acidic nanoparticles prevents doxorubicin cardiotoxicity through improvement of lysosomal function

**DOI:** 10.7150/thno.86310

**Published:** 2023-10-02

**Authors:** Yohan Santin, Karina Formoso, Fraha Haidar, Maria Del Pilar Oreja Fuentes, Florence Bourgailh, Nesrine Hifdi, Karim Hnia, Yosra Doghri, Jessica Resta, Camille Champigny, Séverine Lechevallier, Maximin Détrait, Grégoire Cousin, Malik Bisserier, Angelo Parini, Frank Lezoualc'h, Marc Verelst, Jeanne Mialet-Perez

**Affiliations:** 1 Institute of Metabolic and Cardiovascular Diseases (I2MC), Université de Toulouse, INSERM, Université Toulouse III - Paul Sabatier (UPS), Toulouse, France.; 2 Center for Electron Microscopy Applied to Biology (CMEAB), Université de Toulouse, Faculté de Médecine, Université Toulouse III - Paul Sabatier (UPS), Toulouse, France.; 3 Center for Materials Development and Structural Studies (CEMES), Université de Toulouse, CNRS, Université Toulouse III - Paul Sabatier (UPS), Toulouse, France.; 4 Department of Cardiology, Rangueil University Hospital, Toulouse, France.; 5 New York Medical College, New York, Department of Cell Biology and Anatomy, and of Physiology, Valhalla, New York, United States.

**Keywords:** doxorubicin, lysosomes, nanoparticles, cardiotoxicity, autophagy

## Abstract

Doxorubicin (Dox) is an effective anticancer molecule, but its clinical efficacy is limited by strong cardiotoxic side effects. Lysosomal dysfunction has recently been proposed as a new mechanism of Dox-induced cardiomyopathy. However, to date, there is a paucity of therapeutic approaches capable of restoring lysosomal acidification and function in the heart.

**Methods:** We designed novel poly(lactic-co-glycolic acid) (PLGA)-grafted silica nanoparticles (NPs) and investigated their therapeutic potential in the primary prevention of Dox cardiotoxicity in cardiomyocytes and mice.

**Results:** We showed that NPs-PLGA internalized rapidly in cardiomyocytes and accumulated inside the lysosomes. Mechanistically, NPs-PLGA restored lysosomal acidification in the presence of doxorubicin or bafilomycin A1, thereby improving lysosomal function and autophagic flux. Importantly, NPs-PLGA mitigated Dox-related mitochondrial dysfunction and oxidative stress, two main mechanisms of cardiotoxicity. In vivo, inhalation of NPs-PLGA led to effective and rapid targeting of the myocardium, which prevented Dox-induced adverse remodeling and cardiac dysfunction in mice.

**Conclusion:** Our findings demonstrate a pivotal role for lysosomal dysfunction in Dox-induced cardiomyopathy and highlight for the first time that pulmonary-driven NPs-PLGA administration is a promising strategy against anthracycline cardiotoxicity.

## Introduction

Anthracyclines such as doxorubicin (Dox) are among the most widely used chemotherapeutic agents, with effectiveness in many types of malignancies 1. Nevertheless, their clinical use is limited by dose-dependent cardiotoxicity, which can emerge from a few weeks to several years after therapy completion, thus increasing morbi-mortality among cancer survivors 2. Primary prevention of Dox-induced cardiac damage, i.e., at the time of cancer therapy, could be an optimal approach to reduce the risk of side effects. However, it still remains an unmet clinical need. Dexrazoxane is the only Food and Drug Administration (FDA)-approved drug for preventing Dox cardiotoxicity, but its use has been hampered due to the induction of secondary malignant neoplasms 3.

Though most prior research has focused on DNA damage and mitochondrial dysfunction as the sole mechanisms of Dox cardiac toxicity, recent discoveries put forward a key role for autophagic flux blockade in the etiology of Dox-induced cardiomyopathy 4,5. Autophagy is a dynamic process for lysosome-mediated turnover of damaged mitochondria and proteins, acting as a major cytoprotective mechanism in stress conditions. Autophagy proceeds through sequential steps, consisting in the formation and maturation of autophagosomes and then the fusion with acidic lysosomes to degrade unwanted cytoplasmic constituents. Increasing evidence has highlighted an accumulation of autophagic vesicles in Dox-treated hearts, suggesting that lysosomal impairment might be responsible for the blockade of autophagic flux 6,7. Thus, restoration of lysosomal acidification and function holds promise for the mitigation of Dox cardiotoxicity. However, the lack of specific tools targeting the lysosomes has dramatically impeded the development of such therapeutic strategies.

Interestingly, one original approach relies on the use of nanoparticles that have the advantage of being naturally delivered to lysosomes through endocytic pathways 8. Several studies have employed this property to drive molecules to the lysosomal compartment through nanocarriers, thus facilitating the release of therapeutic agents 9. Indeed, the combination of this unique property of nanoparticles with the use of poly(lactic-co-glycolic acid) (PLGA), which releases glycolic and lactic acids, could rescue lysosomal acidification and function. However, the prospective benefits of such nanoparticles in Dox-dependent cardiac damage are still unknown.

In this study, we designed a new formulation of PLGA-grafted silica nanoparticles (NPs-PLGA) that efficiently targeted the lysosomes and decreased Dox-induced lysosomal alkalinization and dysfunction in cardiomyocytes. Interestingly, NPs-PLGA prevented Dox-related impairments of autophagy and mitigated mitochondrial alterations in both cardiomyocytes and mouse hearts following pulmonary-driven NPs-PLGA administration. Furthermore, our long-term *in vivo* follow-up indicated that NPs-PLGA lessened adverse cardiac remodeling and dysfunction, highlighting the use of acidic nanoparticles as a promising therapeutic strategy for primary prevention of anthracyclines-dependent cardiac damage.

## Results

### Characteristics of PLGA-grafted particles

**Figure 1:**
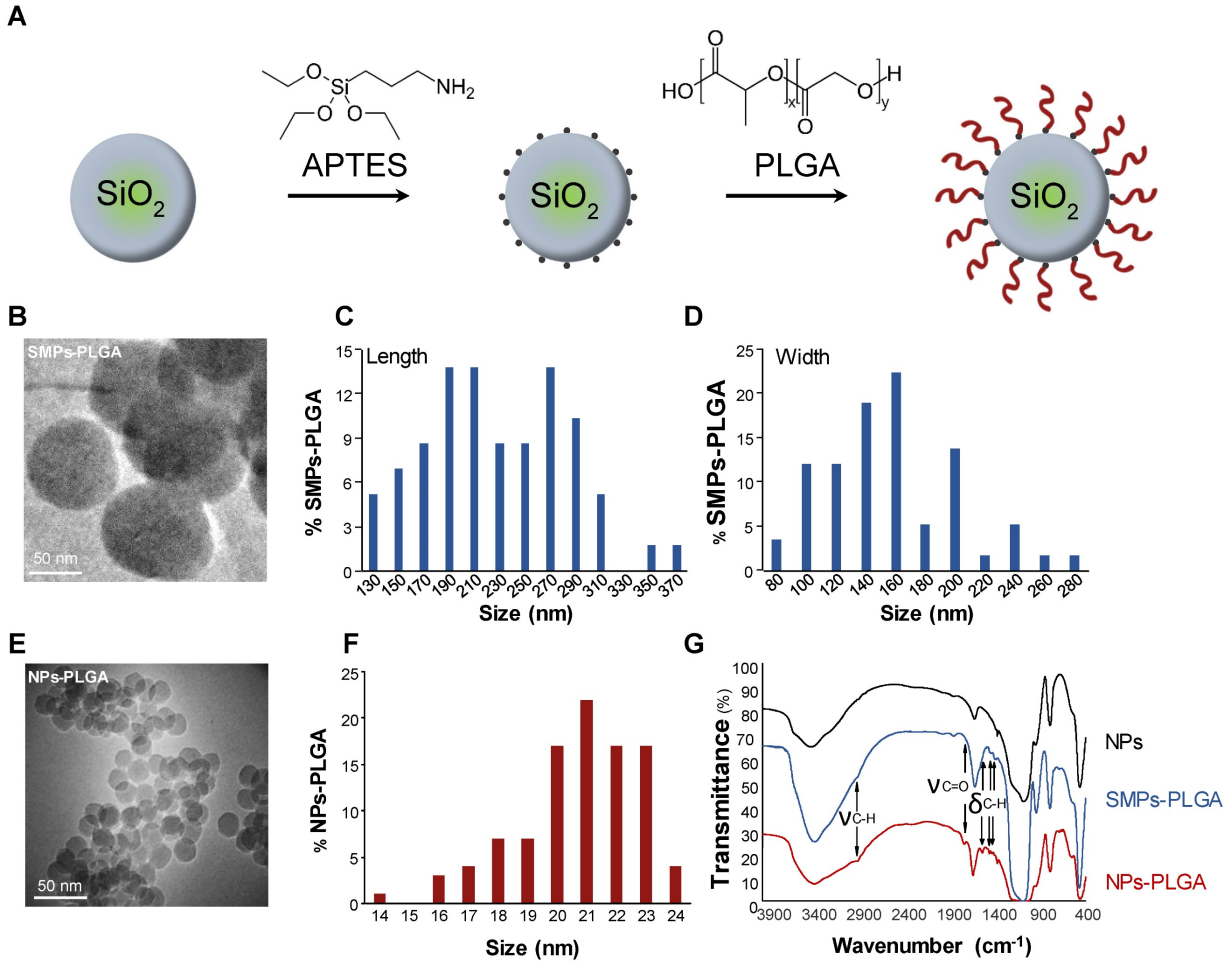
** PLGA-grafted silica particles characterization. (A)** Scheme showing the steps of PLGA grafting on silica particles **(B)** Transmission electron microscopy (TEM) (Scale Bar = 50 nm) and **(C-D)** Size distribution histogram of submicronic PLGA-grafted silica particles (SMPs-PLGA) with **(C)** length and **(D)** width. **(E)** Transmission electron microscopy (TEM) (Scale Bar = 50 nm) and **(F)** Size distribution histogram of PLGA-grafted silica nanoparticles (NPs-PLGA) **(G)** Fourier-transform infrared spectroscopy (FTIR) of NPs, SMPs-PLGA and NPs-PLGA. The x-axis represents the wavenumber (cm^-1^) and the y-axis represents the percentage of transmittance. The spectra of naked particles (NPs) show the presence of well-known Si-O-Si (1070 and 795 cm^-1^), Si-O (461 cm^-1^), and Si-O-H (3422 cm^-1^) bands and a strong peak at 1630 cm^-1^ attributed to water molecules. Additional bands observed on SMPs-PLGA and NPs-PLGA correspond to ν_C-H_ (2945 cm^-1^) and δ_C-H_ (1550, 1473, 1430 cm^-1^) of both APTES and PLGA and to ν_C=O_ of PLGA (1750 cm^-1^).

Silica particles were prepared and characterized as shown in Figure 1. APTES ((3-Aminopropyl) triethoxysilane) was first grafted at the surface of the particles to create hooks and PLGA was then added to previously grafted APTES (Figure 1A). As particles' cellular internalization and downstream functional effects mainly depend on their size, two types of particles were primarily developed. First, submicronic PLGA-grafted silica particles (SMPs-PLGA) were prepared and visualized by transmission electron microscopy (TEM) and scanning electron microscopy (SEM), where they appeared ovoid and poly-dispersed (Figure 1B-D, Figure S1A-B). Primary particles were 238 nm (± 58 nm) in length and 165 nm (± 45 nm) width (Figure 1C-D). Then, smaller silica nanoparticles were grafted with PLGA (nanoparticles NPs-PLGA). The latter appeared spherical and mono-dispersed with an average diameter of 21.3 nm (± 2 nm) (Figure 1E-F). The surface charge of NPs-PLGA was +28.1mV, and the one of SMPs-PLGA was +8 mV, while ζ-potential was -25.6 mV for NPs and -10 mV for SMPs, confirming the efficiency of PLGA grafting. Besides classical bands attributed to silica NPs, the infrared spectra revealed additional bands on SMPs-PLGA and NPs-PLGA, corresponding to ν_C-H_ (2945 cm^-1^) and δ_C-H_ (1550, 1473, 1430 cm^-1^) of both APTES and PLGA and ν_C=O_ of PLGA (1750 cm^-1^) (Figure 1G). Of note, the intensity of C-H and C=O bands were stronger for NPs-PLGA than for SMPS-PLGA, suggesting that the PLGA/SiO_2_ ratio is higher for small nanoparticles (Figure 1G).

### NPs-PLGA are trafficked to lysosomes in cardiomyocytes

**Figure 2:**
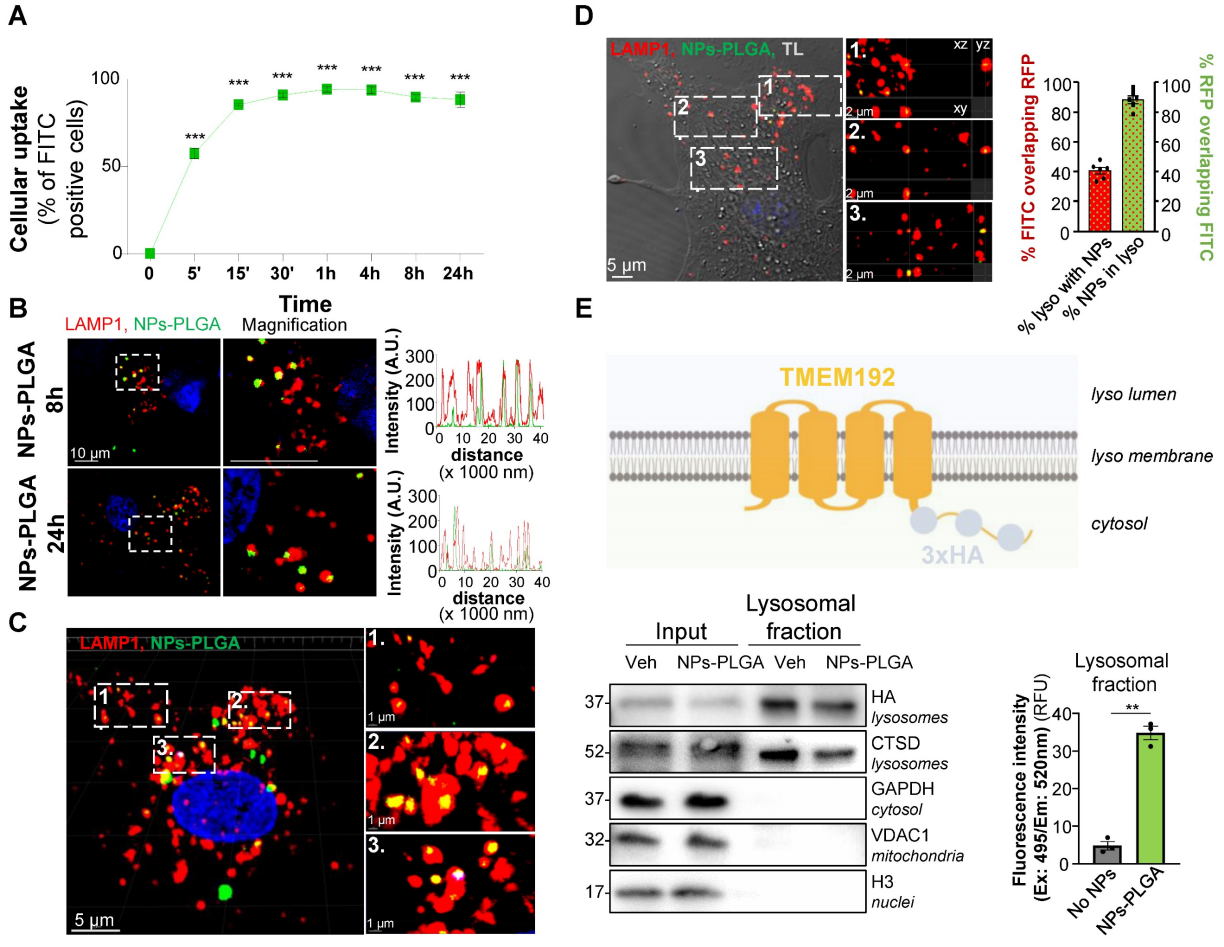
** PLGA-grafted nanoparticles are driven to lysosomal compartment in NRVMs. (A)** Intracellular uptake rate of NPs-PLGA-FITC in NRVMs (n = 3).** (B)** Left panel: Representative confocal images of NRVMs transfected with LAMP1-RFP (red) and incubated with NPs-PLGA-FITC (green) for 8 and 24 h. Hoechst 33258 (blue) was used as counterstaining. Scale Bar = 10 μm. Right panel: Intensity profile graphs of NPs-PLGA-FITC colocalization with LAMP1-RFP.** (C)** 3D reconstruction of stack images of NRVMs transfected with LAMP1-RFP (red) and incubated with NPs-PLGA-FITC (green) for 24 h. Hoechst 33258 (blue) was used as counterstaining. Scale Bar = 5 μm for whole images, 1 µm for magnified areas. **(D)** Left panel: Orthogonal projection of stack images of NRVMs transfected with LAMP1-RFP (red) and incubated with NPs-PLGA-FITC (green) for 24 h. Transmitted light (TL) was used to determine cell boundaries on the whole image and Hoechst 33258 (blue) was used as counterstaining. Scale Bar = 5 μm for whole image, 2 µm for magnified areas. Right panel: Manders' overlap coefficient of green fluorescence overlapping red one and red fluorescence overlapping green one (n = 6).** (E)** Lysosomal immunoisolation in NRVMs transfected with TMEM192-3xHA plasmid. Left panel: Representative immunoblots for protein markers of various subcellular compartments in whole cell lysates (input) and purified lysosomes (lysosomal fraction) from NRVMs incubated or not with NPs-PLGA-FITC. Right panel: quantification of green fluorescence in pure lysosomes from NRVMs incubated or not with NPs-PLGA-FITC (n = 3). Data are expressed as means ± SEM (**p < 0.01, ***p < 0.001 vs t = 0 or No NPs).

Cardiomyocyte uptake of both SMPs-PLGA and NPs-PLGA was then assessed by flow cytometry with particles incorporating fluoresceine isothiocyanate (FITC) for easy detection. Our results showed that SMPs-PLGA exhibited a progressive cellular uptake in Neonatal Rat Ventricular Myocytes (NRVMs), reaching a peak at 8h, but rapidly decreasing between 8h and 24h (Figure S1C). In contrast, NPs-PLGA displayed a more efficient internalization, as up to 90% of cells showed a fast uptake of particles that was sustained until 24h of incubation (Figure 2A). As our goal was to drive those particles to the lysosomes, we assessed their subcellular localization in cardiac cells transfected with a LAMP1-RFP construct. Importantly, confocal imaging showed that SMPs-PLGA appeared randomly dispersed in the cells at 8h and 24h, and did not reach the lysosomes (Figure S1D). On the contrary, NPs-PLGA revealed a noteworthy lysosomal localization, as demonstrated by the strong colocalization on intensity profile graphs between NPs-PLGA fluorescence and lysosomal staining (LAMP1-RFP) at 8h and 24h (Figure 2B). Interestingly, 3D-reconstruction and orthographic projection of confocal images supported that SMPs-PLGA localized sporadically in the cytoplasm (Figure S1E-F), while NPs-PLGA appeared as yellow puncta surrounded by LAMP1-RFP^+^ organelles in NRVMs (Figure 2C-D). Furthermore, Manders' overlap coefficient showed that around 90% of intracellular NPs-PLGA were localized in lysosomes. In comparison, 40% of lysosomes exhibited NPs-PLGA uptake (Figure 2D). Finally, we performed lysosome immunoisolation using HA-magnetic beads in NRVMs expressing transmembrane Protein 192 (TMEM192) fused to 3 tandem HA-epitopes, as previously described 10 (Figure 2E). Our results show that lysosomes purified from NPs-PLGA-treated cells exhibited a notable increase in FITC fluorescence compared to lysosomes from non-treated NRVMs, which confirms the presence of NPs-PLGA within these organelles (Figure 2E). Of note, cellular uptake and lysosomal addressing of NPs-PLGA were further confirmed in the H9C2 cardiomyoblast cell line (Figure S2A-D).

Altogether, these data suggest that PLGA-grafted silica nanoparticles of size around 20 nm are efficiently driven to lysosomes in cardiomyocytes.

### NPs-PLGA prevent Bafilomycin A1- and Doxorubicin-induced lysosomal alterations

**Figure 3:**
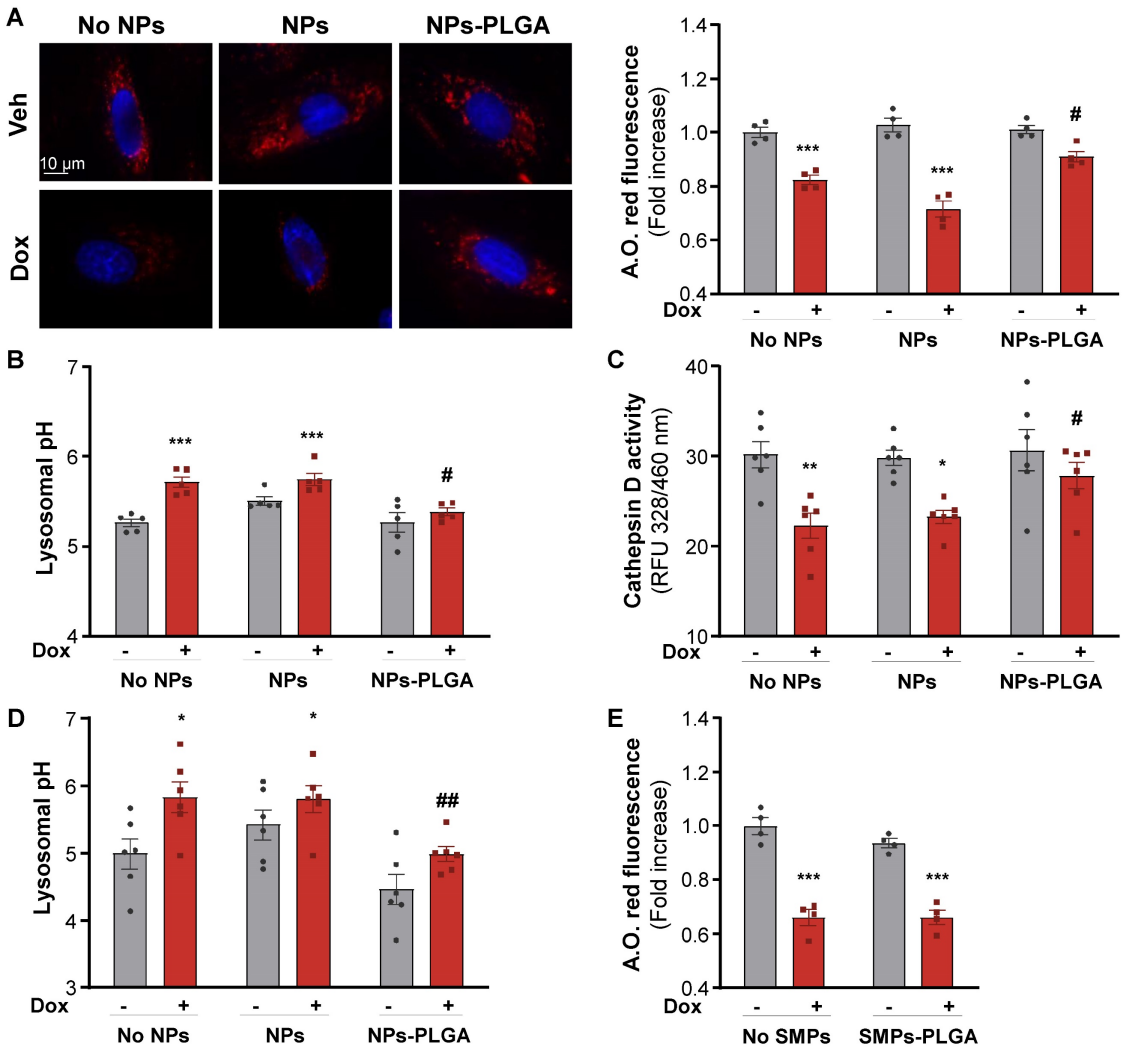
** NPs-PLGA prevent Dox-induced lysosomal dysfunction. (A-C)** H9C2 were pre-incubated O/N with Veh or 25 µg/mL of NPs or NPs-PLGA and treated with doxorubicin (1 µM, 10 h).** (A)** Left panel: Representative images of Acridine Orange red fluorescence on H9C2. Hoechst 33258 (blue) was used as counterstaining. Scale Bar = 10 µm. Right panel: Fluorimeter measurements of Acridine Orange red staining (n = 4). **(B)** Fluorimeter measurements of Lysosensor yellow/blue staining on H9C2 (n = 5). Excitation was measured at 360 nm and the ratio of emission 440/540 nm was calculated. **(C)** Fluorimeter measurements of Cathepsin D activity fluorescence on H9C2 (n = 6)** (D-E)** NRVMs were pre-incubated O/N with Veh or 200 µg/mL of NPs, SMPs, NPs-PLGA or SMPs-PLGA and treated with doxorubicin (1 µM, 10 h).** (D)** Fluorimeter measurements of Lysosensor yellow/blue staining on NRVMs (n = 6). **(E)** Fluorimeter measurements of Acridine Orange red staining on NRVMs (n = 4). Data are expressed as means ± SEM (*p < 0.05, **p < 0.01, ***p < 0.001 vs Veh; #p < 0.05, ##p < 0.01 vs Dox).

Given their intra-lysosomal localization, we investigated whether NPs-PLGA could influence lysosomal pH and function. In a 20 mM PBS solution at pH 6, designed to simulate the lysosomal environment, NPs-PLGA were able to acidify the solution within the first 12h of incubation, while no acidification was observed when NPs-PLGA were exposed to a pH 7.4 PBS solution (Figure S3A). We then evaluated NPs-PLGA innocuity by measuring cell death in H9C2 cells, and found that NPs-PLGA were safe until 25 µg/mL (Figure S3B). NPs-PLGA safety was confirmed by the absence of apoptosis induction at 25 µg/mL and lower concentrations (Figure S3C). Next, we treated H9C2 cells with Bafilomycin A1 (Baf A1), a potent inhibitor of lysosomal acidification. Interestingly, pre-incubation of cells with NPs-PLGA (25 µg/mL) significantly prevented the drop of acridine orange fluorescence (a metachromatic dye that accumulates in acidic vesicles of living cells) induced by Baf A1, while silica nanoparticles devoid of PLGA (NPs) had no effect (Figure S3D). In addition, measurement of lysosomal pH indicated that NPs-PLGA increased lysosomal acidification, as shown by a decrease in lysosomal pH in the presence of Baf A1, while ungrafted NPs had no effect (Figure S3E). Then, we addressed the effects of NPs-PLGA on Dox-induced lysosomal alkalinization, and found that NPs-PLGA prevented the loss of acridine orange fluorescence induced by Dox in H9C2 cells (Figure 3A). Consistently, Dox increased lysosomal pH and led to the alteration of lysosomal proteolysis, assessed by a decrease in Cathepsin D activity, while both effects were prevented by NPs-PLGA (Figure 3B, C). Importantly, the effect of NPs-PLGA on lysosomal pH was further assessed in NRVMs, where they did not show any toxicity even at high doses (Figure S4A). Similar to H9C2 cells, NPs-PLGA (200 µg/mL) were effective to acidify the lysosomes in the presence of Dox, while NPs without PLGA had no effect (Figure 3D, Figure S4B). Of note, likely due to extra-lysosomal distribution, SMPs-PLGA did not exert any improvement on Dox-induced lysosomal alkalinization (Figure 3E).

Altogether, these results indicate that intra-lysosomal PLGA improves lysosomal pH and function in the presence of the alkalinizing agents Baf A1 and Dox.

### Lysosomal NPs-PLGA prevent Dox-induced cardiac autophagy dysfunction

**Figure 4:**
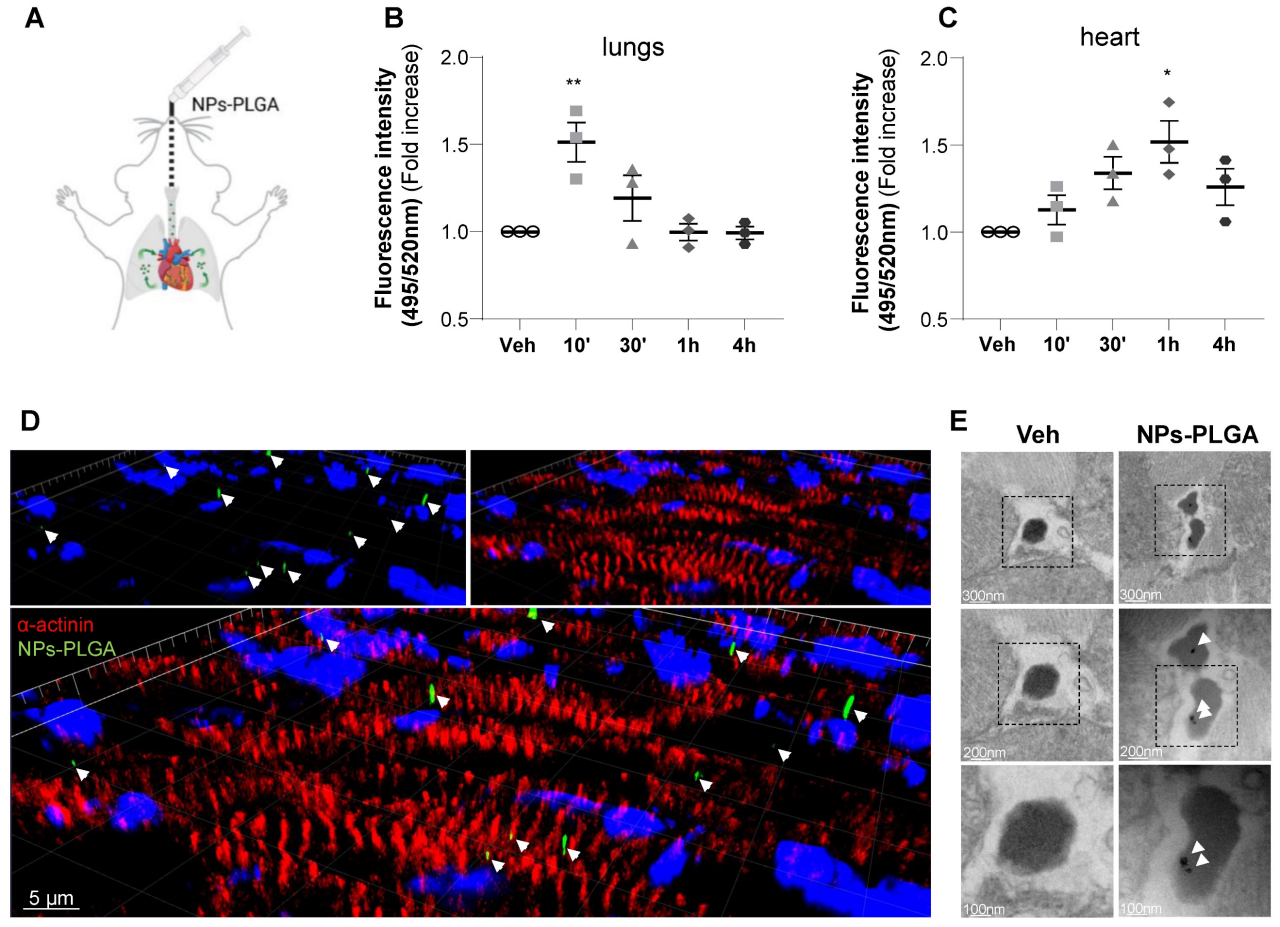
** Intratracheal administration of NPs-PLGA leads to cardiac lysosomes addressing in mice. (A)** Schematic representation of NPs-PLGA administration via intratracheal nebulization in mice. **(B-C)** Fluorescence of NPs-PLGA-FITC in (B) lungs and (C) hearts of mice treated with Veh or 1 mg/kg of NPs-PLGA-FITC through intratracheal nebulization for 10', 30', 1 h or 4 h. Data are normalized per mg of tissue. (n = 3) **(D)** 3D reconstruction of stack images of heart tissues from mice collected 1 h after intratracheal nebulization of Veh or 1 mg/kg of NPs-PLGA-FITC. Green: NPs-PLGA-FITC, red: alpha-actinin, blue: DAPI. Scale bar = 5 µm. **(E)** Transmission Electron Microscopy of cardiac lysosomes from mice after 1h of Veh or NPs-PLGA-FITC (1 mg/kg) administration via intratracheal nebulization. Data are expressed as means ± SEM (*p < 0.05, **p < 0.01 vs Veh).

To assess the therapeutic potential of nanoparticles in the myocardium, NPs-PLGA incorporating FITC were administered through the lungs using intratracheal nebulization in mice (Figure 4A). Analysis of tissue distribution showed that NPs-PLGA fluorescence increased rapidly in mouse lungs, followed by a decrease that paralleled myocardial accumulation, suggesting a transfer of NPs-PLGA from the pulmonary circulation to the heart (Figure 4B-C, Figure S5A-E). These results were further supported by immunostaining experiments on cardiac cryosections, as 3D reconstruction and orthogonal projection of confocal images highlighted an effective targeting of NPs-PLGA in cardiac tissues 1h post-administration (Figure 4D, Figure S5F). Importantly, electron microscopy analysis of ventricular tissues revealed the presence of nanoparticles characterized as electron-dense structures into lysosomal-related vesicles based on ultrastructural parameters, compared to untreated heart samples (Figure 4E). These observations suggest that intratracheally-administered NPs-PLGA are driven to cardiac lysosomes.

**Figure 5:**
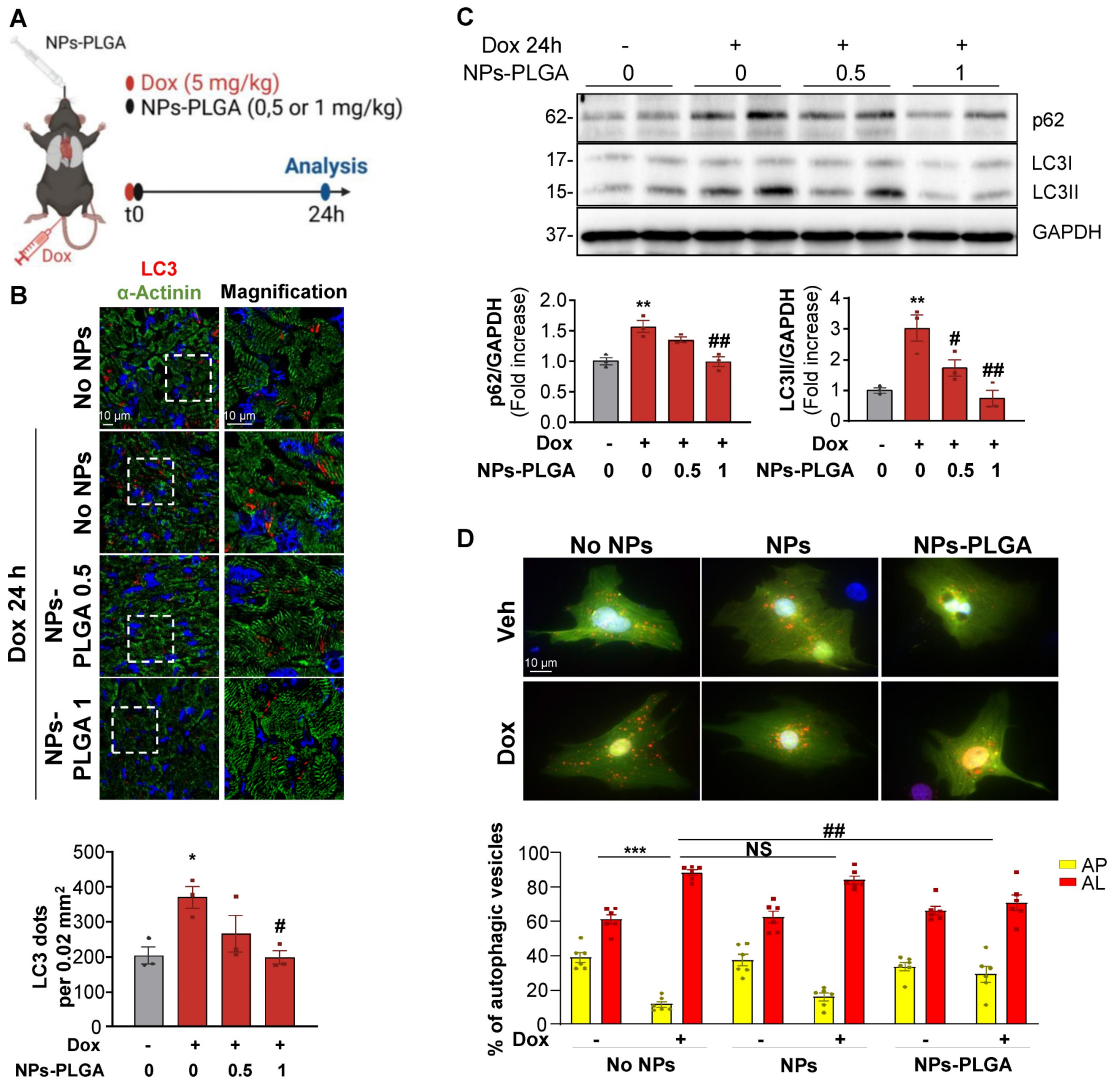
** NPs-PLGA avoid autophagy alterations after Dox treatment. (A)** Schematic representation of the experimental design in mice treated with Veh or Dox (5 mg/kg) ± NPs-PLGA (0.5 or 1 mg/kg) for 24 h. Dox and NPs-PLGA were administered at the same time. **(B)** Upper panel: Representative confocal images of heart tissues from mice 24 h after Veh or Dox (5 mg/kg) ± NPs-PLGA (0.5 or 1 mg/kg) administration. Green: alpha-actinin, red: LC3, blue: DAPI. Scale bar = 10 µm. Lower panel: Quantification of the number of LC3-positive dots per 0.02 mm^2^ (n = 3). **(C)** Upper panel: Representative immunoblots and lower panel: quantifications of p62 and LC3II expression in cardiac homogenates of mice 24 h after Veh or Dox (5 mg/kg) ± NPs-PLGA (0.5 or 1 mg/kg) administration (n = 3). GAPDH was used as loading control. **(D)** Upper panel: Double immunofluorescence imaging of RFP-GFP-LC3-transfected NRVMs pre-incubated O/N with Veh or 200 µg/mL of NPs or NPs-PLGA and treated with doxorubicin (1 µM, 16 h). Scale bar = 10 µm. Lower panel: Quantification of yellow puncta (AP, autophagosomes) and red puncta (AL, autolysosomes) for each condition (n = 6). Data are expressed as means ± SEM (*p < 0.05, **p < 0.01 vs Veh; #p < 0.05, ##p < 0.01 vs Dox).

Considering the beneficial role of NPs-PLGA on lysosomal function, and since lysosomes play a key role in autophagy completion, we then assessed the effects of NPs-PLGA on autophagy in Dox-treated mice (Figure 5A). Immunofluorescence staining showed that Dox elicited an accumulation of LC3-positive cargos in mouse hearts after 24h. This effect was prevented by intratracheal administration of increasing doses of NPs-PLGA, given at the same time as Dox (Figure 5B). Accordingly, accumulation of the autophagic markers LC3II and p62 in Dox-treated mouse ventricles was mitigated by intratracheal administration of NPs-PLGA in a dose-dependent manner (Figure 5C). Considering the role of NPs-PLGA on lysosomal improvement, these results suggest that prevention of LC3II and p62 accumulation is likely due to improved clearance of autophagic cargos by functional lysosomes rather than a decrease of autophagosomes formation. To test this assumption, we used a GFP-RFP-LC3 reporter construct *in vitro* to visualize autophagosomes in yellow (GFP^+^RFP^+^) and autolysosomes in red (GFP^-^RFP^+^). As a consequence of lysosomal alkalinization, Dox induced an accumulation of autolysosomes, an event mitigated by NPs-PLGA but not NPs in NRVMs and H9C2 cells (Figure 5D, Figure S6A). To further support the benefits of NPs-PLGA on autophagic clearance, autophagic flux was assessed in the presence of Baf A1. Consistently, Baf A1-induced blockade of autophagic flux was mitigated by NPs-PLGA, as indicated by the smaller accumulation of GFP^+^RFP^+^ vesicles (Figure S6B) and lower LC3II and p62 protein levels (Figure S6C).

Altogether, these data support the beneficial role of NPs-PLGA in the improvement of Dox-impaired autophagic flux in cardiac cells.

### NPs-PLGA-mediated autophagic clearance decreases Dox-induced mitochondrial dysfunction

**Figure 6:**
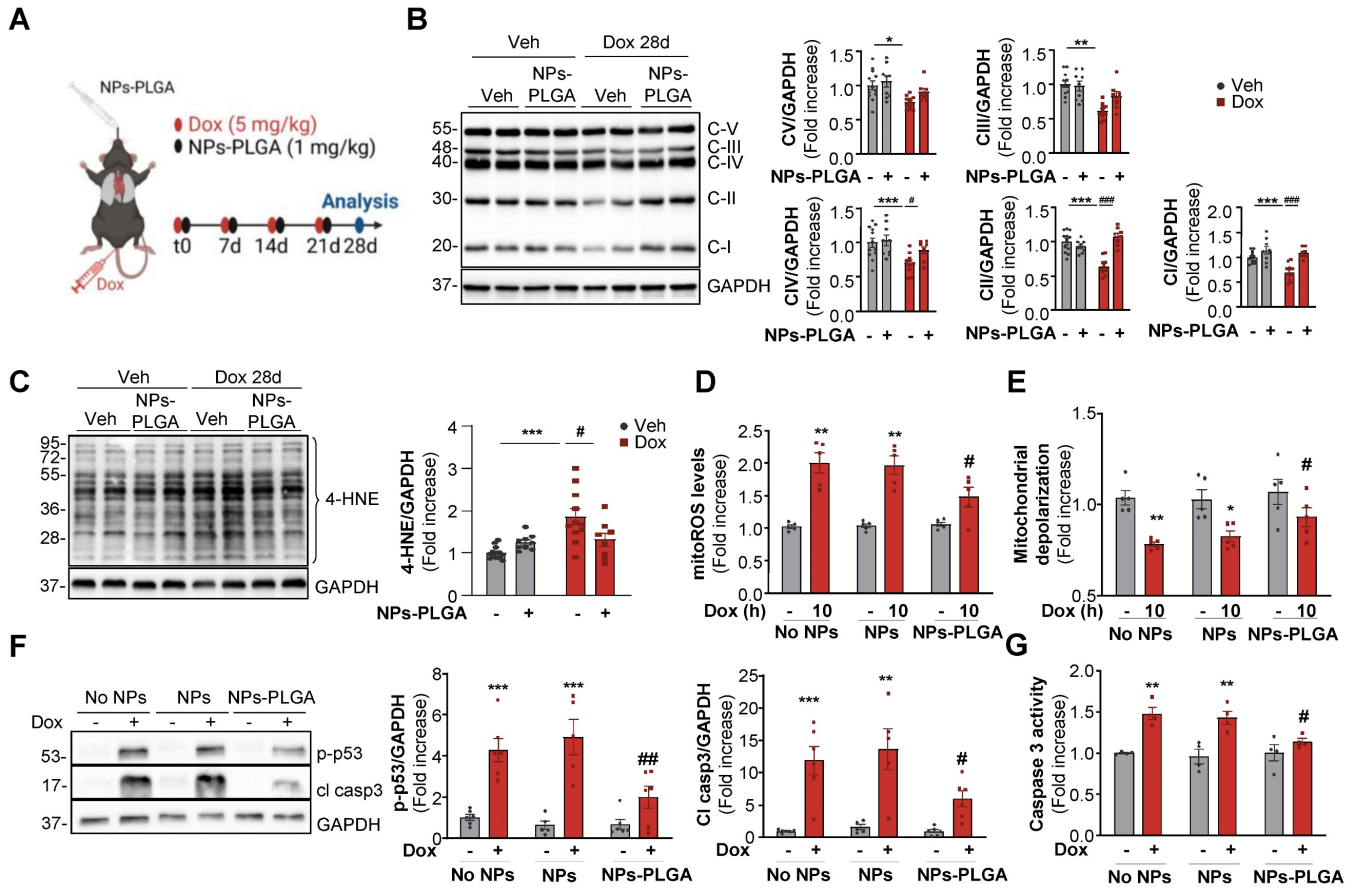
** NPs-PLGA limit Dox-induced mitochondrial dysfunction. (A)** Experimental design in mice treated 4 times with once-per-week injections of Veh or Dox (5 mg/kg) ± NPs-PLGA (1 mg/kg) at the same time, and analysed at 28 days. **(B-C)** Immunoblots from cardiac homogenates of mice at 28 d with:** (B)** OXPHOS complexes (Left panel: Representative immunoblots and right panel: quantifications) (n = 8-12). GAPDH was used as a loading control.** (C)** 4-HNE (Left panel: Representative immunoblots and right panel: quantifications) (n = 8-12). GAPDH was used as a loading control. **(D-F)** H9C2 were pre-incubated O/N with Veh or 25 µg/mL of NPs or NPs-PLGA, and treated with Veh or Dox (1 µM). **(D)** Quantification of mitoROS assessed by mitoSOX fluorescence on H9C2 treated with Veh or Dox (1 µM, 10 h) (n = 5)** (E)** Quantification of mitochondrial depolarization assessed by fluorescence of JC-1 red aggregates/green monomers ratio on H9C2 treated with Veh or Dox (1 µM, 10 h) (n = 5) **(F)** Left panel: Representative immunoblots and right panel: quantifications of p-p53 and cleaved caspase 3 protein expression on H9C2 treated with Veh or Dox (1 µM, 24 h). GAPDH was used as a loading control. (n=6) **(G)** Quantification of caspase 3 activity in NRVMs treated with Veh or Dox (1 µM, 24 h) (n = 4). Data are expressed as means ± SEM (*p < 0.05, **p < 0.01, ***p < 0.001 vs Veh; #p < 0.05, ##p < 0.01, ###p < 0.001 vs Dox).

Mitochondrial dysfunction is a crucial determinant of Dox cardiotoxicity. The removal of damaged mitochondria is mainly achieved through autophagy and is thus critical for maintaining proper cardiomyocyte function. As mitochondria are key players in the generation and regulation of cellular bioenergetics, we primarily assessed the oxidative phosphorylation system (OXPHOS) in cardiac ventricles of mice treated with Dox. In this goal, we employed a preclinical model that mimicked the clinical protocol in patients 7, which consisted of 4 once-weekly injections of low-dose doxorubicin (5 mg/kg) in mice (Figure 6A). Our results showed that one single administration of Dox as described in Figure 5A triggered an early impairment of mitochondrial OXPHOS (Figure S7A) and these alterations were still visible in the longer term after 4 weekly Dox injections (Figure 6B). Interestingly, NPs-PLGA did not prevent early defects but mitigated long-term mitochondrial alterations (Figure S7A, Figure 6B). As dysfunctional mitochondria are major sources of oxidative stress, we then measured the levels of 4-hydroxynonenal (4-HNE), a stable end-product of ROS-mediated lipid peroxidation, in ventricular homogenates of mice after 4 weekly Dox injections (Figure 6C). NPs-PLGA significantly decreased the accumulation of 4-HNE in cardiac homogenates of Dox-treated mice (Figure 6C). Consistenly, Dox-induced mitoROS accumulation in H9C2 cells was prevented by NPs-PLGA after long-term Dox treatment (10 h) contrary to shorter times (4 h) (Figure 6D, Figure S7B). Accordingly, Dox-induced mitochondrial depolarization was mitigated by NPs-PLGA after long-term Dox treatment (10 h) (Figure 6E). These results suggest that NPs-PLGA long-term benefits are likely due to improvement in mitochondrial clearance by autophagy rather than a direct effect on mitochondrial function. Importantly, the benefits of lysosomal acidification were further supported by the counteracting effects of NPs-PLGA on Dox-activated pro-apoptotic factors p-p53 and cleaved-caspase 3 (Figure 6F-G).

Overall, our results show that Dox-induced lysosomal dysfunction is at the cornerstone of Dox cardiotoxicity, as amelioration of lysosomal pH by NPs-PLGA improves autophagic flux, mitochondrial function, and cell viability.

### NPs-PLGA prevent adverse cardiac remodeling and dysfunction in Dox-treated mice

**Figure 7:**
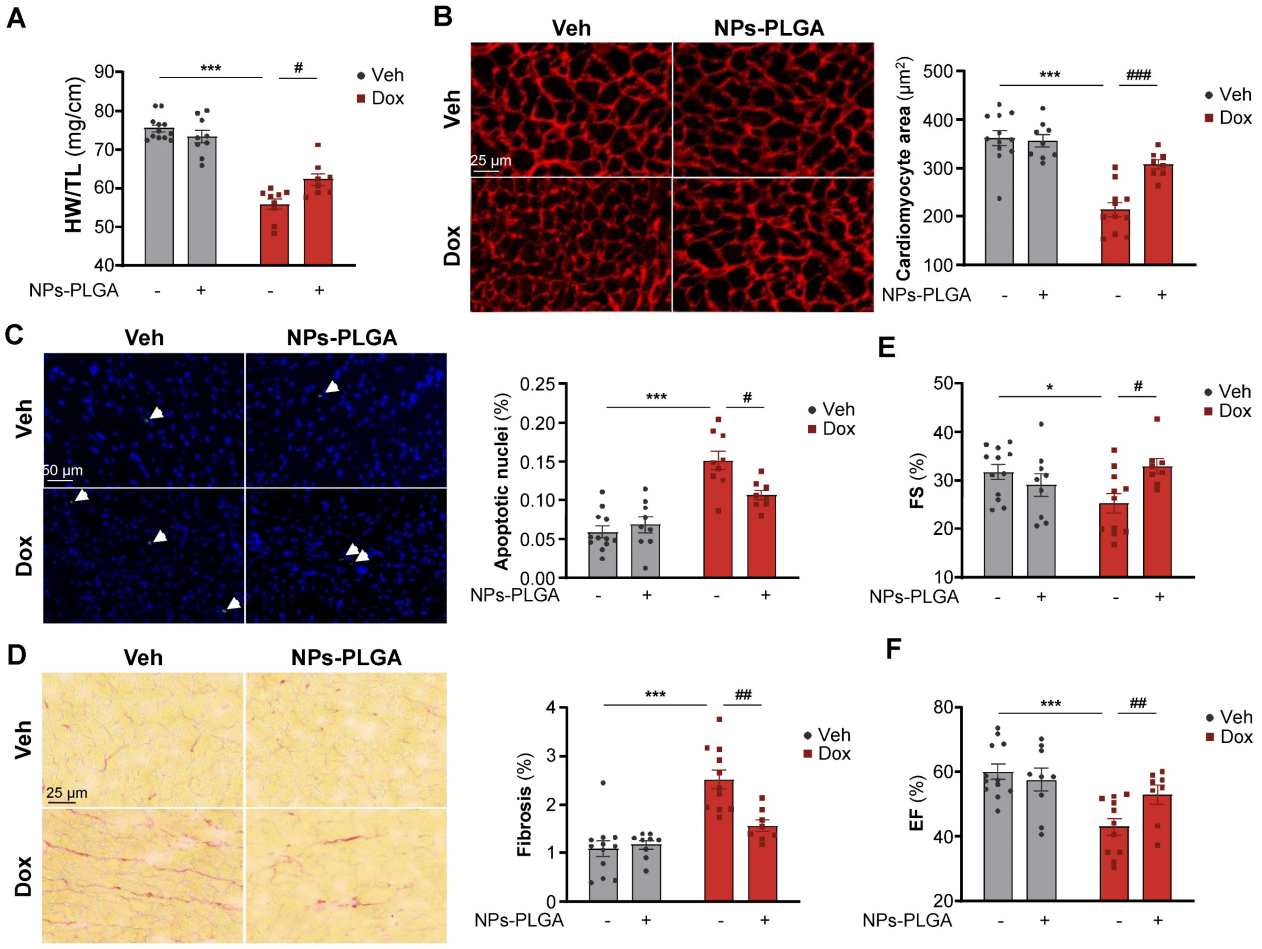
** Dox-induced cardiac adverse remodeling and dysfunction are prevented upon NPs-PLGA treatment. (A-F)** Evaluation of cardiac parameters after 4 once-per-week injections of Veh or Dox (5 mg/kg) ± NPs-PLGA (1 mg/kg). Dox and NPs-PLGA were administered at the same time. **(A)** Cardiac remodeling was evaluated by determining the Heart weight-to-Tibia length ratio (n = 8-12).** (B)** Left panel: Representative images of Vinculin staining (Scale bar = 25 µm) and right panel: quantification of cardiomyocyte area in heart sections (n = 8-12). **(C)** Left panel: Representative images of TUNEL staining (Scale bar = 50 µm) and right panel: quantification of TUNEL positive nuclei in heart sections (n = 8-12). **(D)** Left panel: Representative images of Sirius Red staining (Scale bar = 25 µm) and right panel: quantification of collagen content in heart sections (n = 8-12). **(E-F)** Cardiac function was assessed by echocardiographic parameters with **(E)** Fractional Shortening (n = 8-12) and **(F)** Ejection Fraction (n = 8-12). Data are expressed as means ± SEM (*p < 0.05, ***p < 0.001 vs Veh; #p < 0.05, ##p < 0.01, ###p < 0.001 vs Dox).

To investigate the therapeutic benefits of inhaled NPs-PLGA on the development of Dox-related cardiomyopathy at 5 weeks, we treated mice with 4 once-weekly injections of low-dose doxorubicin (5 mg/kg) according to the protocol described in Figure 6A. Of note, the inhalation protocol did not induce lung congestion indicating that pulmonary-administered NPs-PLGA did not alter respiratory function in mice (Figure S8A). Most interestingly, Dox-treated mice exhibited reduced heart weight (HW) and ventricular weight (VW), and these effects were limited by the administration of NPs-PLGA (Figure 7A, Figure S8B). As cardiac mass loss mainly results from cardiomyocyte atrophy and death, we then measured cell size and apoptosis in hearts of Dox-treated mice (Figure 7B-C). Our results showed that cardiomyocyte atrophy induced by Dox was prevented by NPs-PLGA (Figure 7B). Besides, the increase of TUNEL-positive nuclei induced by Dox was lessened by NPs-PLGA indicating a prevention of apoptosis (Figure 7C). Cardiac fibrosis, another hallmark of Dox cardiotoxicity, was also significantly decreased by NPs-PLGA (Figure 7D). Finally, prevention of Dox-induced cardiac adverse remodeling by NPs-PLGA greatly limited cardiac dysfunction, as shown by the improved fractional shortening and ejection fraction (Figure 7E-F).

Altogether these results demonstrate the cardioprotective effects of intratracheal nebulization of NPs-PLGA against Dox cardiotoxicity.

### NPs-PLGA do not impair Dox-mediated cytotoxic effects on cancer cells

**Figure 8:**
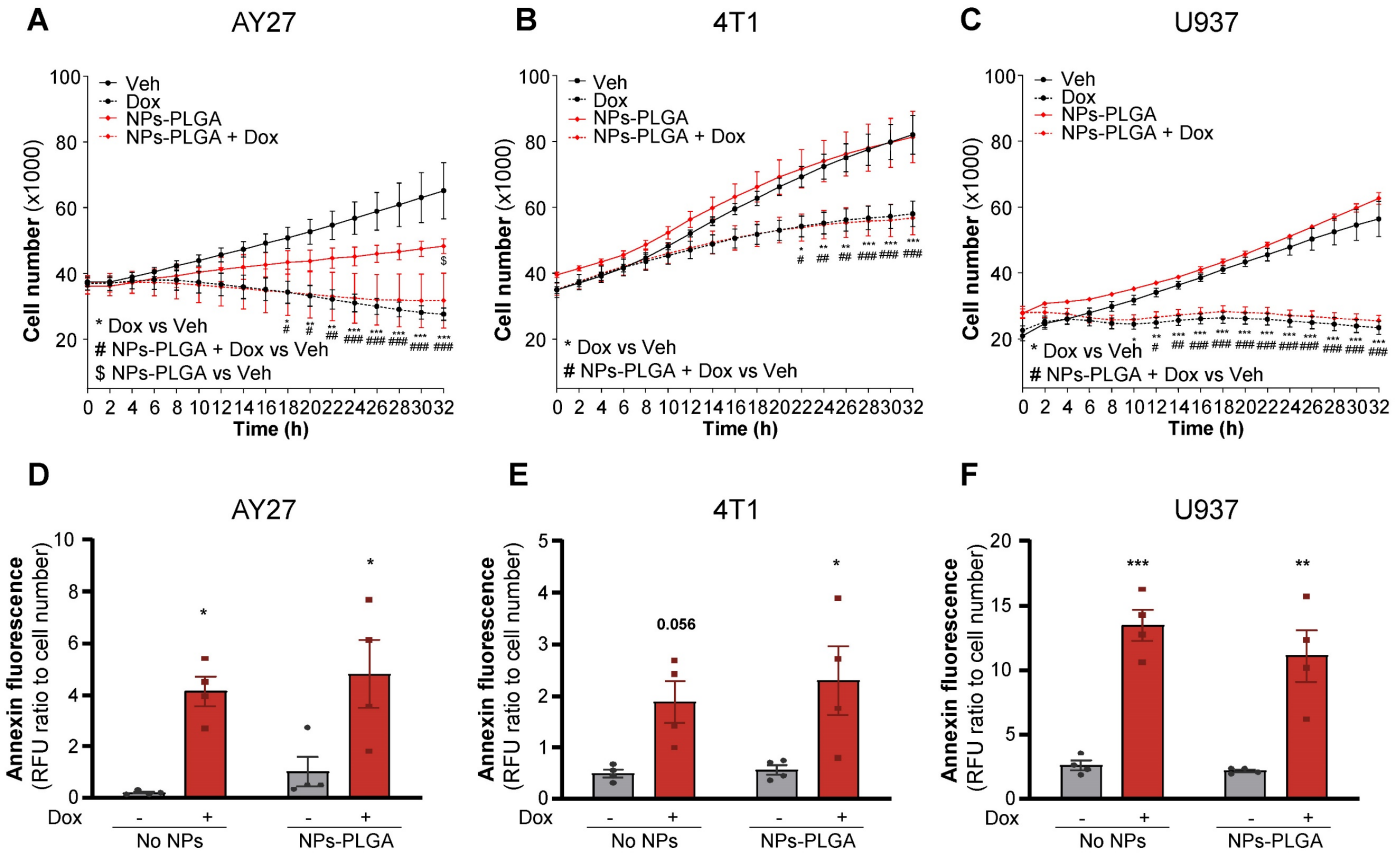
** NPs-PLGA do not alter Dox anti-cancer effect. (A-C)** Proliferation curves of cancer cells incubated with Veh or NPs-PLGA (200 µg/mL) and treated with Veh or Dox (5 µM) with **(A)** rat urothelial carcinoma AY27 cells** (B)** mouse mammary carcinoma 4T1 cells and **(C)** human histiocytic lymphoma U937 cells (n = 4).** (D-F)** Apoptosis measured by annexin fluorescence normalized to cell number in cancer cells incubated with Veh or NPs-PLGA (200 µg/mL) and treated with Veh or Dox (5 µM) with **(D)** rat urothelial carcinoma AY27 cells** (E)** mouse mammary carcinoma 4T1 cells and **(F)** human histiocytic lymphoma U937 cells (n = 4). Data are expressed as means ± SEM (*p < 0.05, **p < 0.01, ***p < 0.001 for Dox vs Veh, #p < 0.05, ##p < 0.01, ###p < 0.001 for NPs-PLGA+Dox vs Veh, $p < 0.05 for NPs-PLGA vs Veh).

Finally, to determine if Dox chemotherapeutic properties could be affected by NPs-PLGA, we assessed their effects in various types of cancer cells. In this goal, rat urothelial carcinoma AY27 cells, mouse mammary carcinoma 4T1 cells, and human histiocytic lymphoma U937 cells were exposed to Dox in the presence or absence of NPs-PLGA (Figure 8). As expected, Dox strongly inhibited the proliferation of all tumor cell lines and this effect was not modified by NPs-PLGA (Figure 8A-C). It is interesting to note that in AY27 urothelial cells, NPs-PLGA *per se* limited cell proliferation (Figure 8A). We next measured annexin fluorescence in the same conditions, and we observed that NPs-PLGA did not alter Dox-induced apoptosis in AY27, 4T1 and U937 cells (Figure 8D-F). These results suggest that NPs-PLGA can limit Dox cardiotoxicity without impairing its anticancer effects, further supporting their therapeutic potential in Dox-induced cardiac damage.

## Discussion

**Figure 9:**
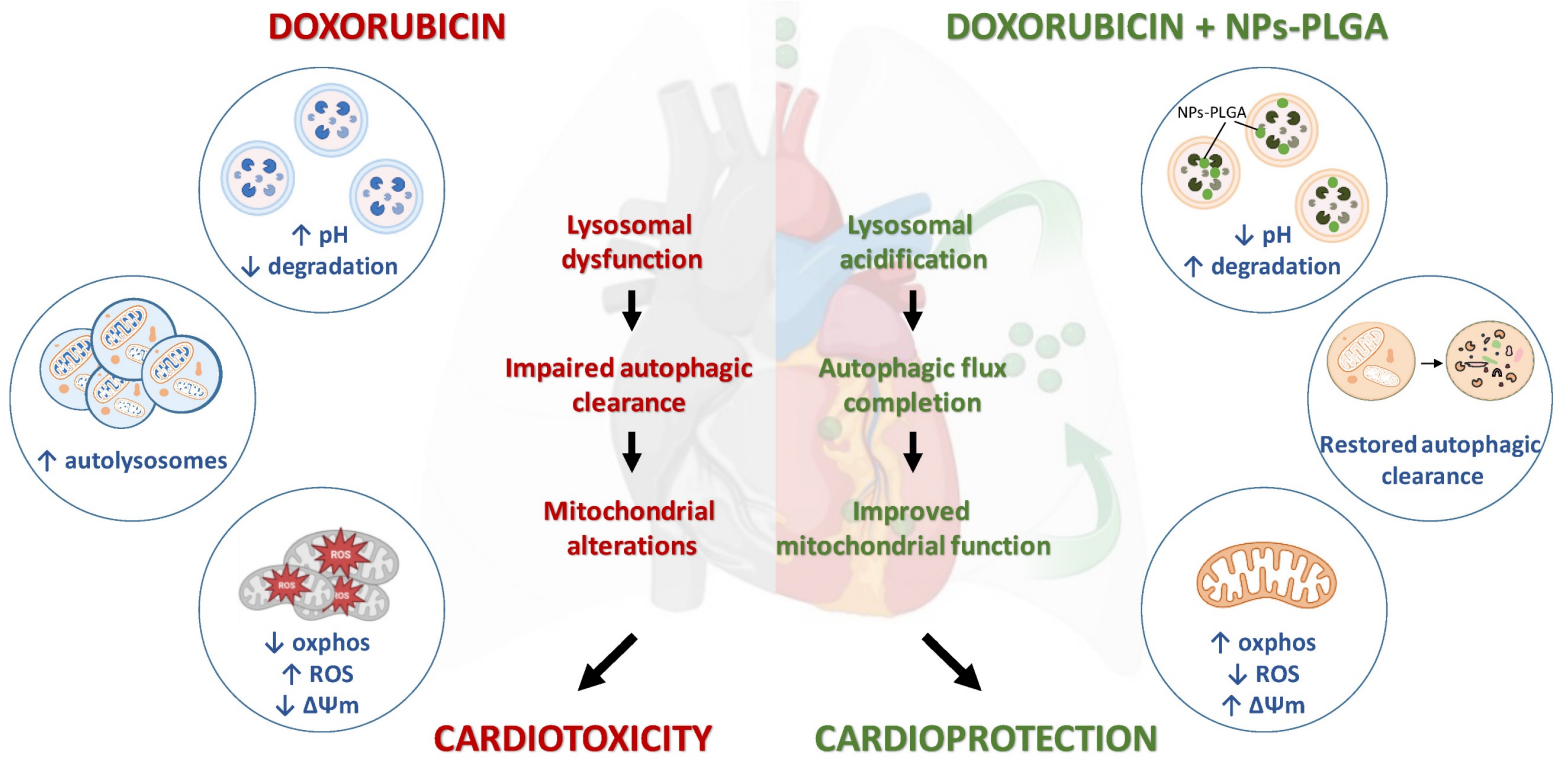
** Role of lysosomal dysfunction and benefits of NPs-PLGA in Dox cardiotoxicity.** Dox induces an increase in lysosomal pH, contributing to organelle dysfunction, autophagic flux blockade and exacerbating mitochondrial alterations, all of these participating in cardiotoxicity. Co-administration of NPs-PLGA with Dox prevents lysosomal alkalinization through restoration of lysosomal acidity, thereby restoring autophagic flux and preserving mitochondria function, leading to cardioprotection. This figure was created with BioRender.com.

Although doxorubicin cardiomyopathy has been extensively studied, its underlying molecular mechanisms are still not fully understood. Dysregulation of cardiac autophagy has been implicated in anthracycline cardiotoxicity but with conflicting findings 4,11. In this context, few therapeutic strategies have been tested for improving autophagy in Dox cardiomyopathy 12. Here, we demonstrate the pivotal role of the disruption of lysosomal pH and function in the onset of Dox-induced cardiomyopathy, and we propose intratracheal nebulization of PLGA-grafted silica nanoparticles as an effective therapeutic strategy to reacidify lysosomes and thus limit Dox‐induced cardiac damage (Figure 9).

Nanoparticles prepared by emulsification or nanoprecipitation of PLGA have been widely used in drug delivery systems 13. This kind of nanotechnology relies on the enfolding of pharmacological agents within PLGA agglomerates, so the therapeutic effects belong to the released drugs but do not depend on PLGA itself 14. Nevertheless, PLGA nanoparticles have also shown intriguing benefits in various disease models for the restoration of lysosomal acidification defects, with positive outcomes on functional parameters 15-18. Polymeric PLGA nanoparticles offer great biodegradability but exhibit complexities in manufacturing processes that affect their size and morphology 19. These recent findings prompted us to develop a novel formulation of silica NPs with specific features to promote optimal lysosomal uptake while also leveraging the acidification properties of PLGA. As silica NPs have been broadly used for biomedical applications in diagnosis and therapy, we designed finely tuned silica-core particles of two different sizes. Silica NPs exhibit various advantages, such as chemical stability, easy synthesis, and functionalization, as well as good biocompatibility 20. These two types of particles were then grafted with PLGA polymers on their surface, allowing the slow release of acidic functions by hydrolysis of the two corresponding organic acids, glycolic and lactic acids. Our data show that contrary to SMPs-PLGA (≈ 240 nm) that exhibit a transitory cellular uptake and localize sporadically in the cytoplasm, NPs-PLGA (≈ 20 nm) have a fast and sustained cellular uptake and ultimately reach the lysosomal compartment in cardiac cells. These data confirm previous works where nanoparticles between 20 and 55 nm displayed optimal endocytosis 21,22. In addition, Li et al. showed that particles over 174 nm are randomly dispersed in the cytoplasm and are more cytotoxic, whereas smaller nanoparticles are safely transported and internalized into lysosomes 23. The accumulation of nanoparticles in lysosomes has long been a subject of investigation, with several studies highlighting potential adverse effects known as lysosomal nanotoxicity 24. However, NPs' possible toxicity greatly depends on their specific physicochemical properties, such as shape, size, composition, surface charge, and functionalization 24. For instance, gold NPs have been shown to impair lysosomal activity resulting in impaired autophagic flux, unlike silica nanoparticles 25. Accordingly, our data indicate that NPs-PLGA do not exhibit any cardiotoxic effect until the dose of 50 μg/mL. In addition, we found that intralysosomal NPs-PLGA lessened lysosomal pH alterations in the presence of both bafilomycin A1 and doxorubicin, suggesting that PLGA grafted on silica nanoparticles is likely to have an effective and sustained acidification effect in the lysosomal lumen. Consequently, autophagy defects were prevented, as shown by the inhibition of autophagic vesicles accumulation and the normalization of the autophagic markers LC3II and p62 in cardiac cells. Importantly, these findings suggest that autophagic deregulation induced by Dox is mostly due to lysosomal alterations, which trigger an impaired clearance of autolysosomes. Besides, we showed that amelioration of lysosomal pH by NPs-PLGA correlated with the preservation of mitochondrial function and improved cell viability. Autophagy maintains mitochondrial quality by selectively degrading dysfunctional organelles. In our study, NPs-PLGA-mediated improvement of autophagic flux may underly the restoration of mitochondrial quality control.

Autophagic flux blockade seems to be increasingly recognized as a central mechanism of Dox cardiotoxicity, and altered degradation of autophagic vesicles has been widely reported 6,7,26,27. Interestingly, Li et al. proposed that Dox primarily alters lysosomal function through V-ATPase impairment, thereby compromising lysosomal pH and suppressing autophagic flux 7. To counteract Dox-induced autophagy alterations, the authors decreased autophagy initiation through Beclin 1 haploinsufficiency to reduce the demand on altered lysosomes, allowing more time for the autolysosomes to process their cargo 7. Another strategy employed by Bartlett et al. was to stimulate lysosomal biogenesis and autophagy through gene-transduction of transcription factor EB (TFEB) 26, which has proven benefits in distinct models of cardiac diseases characterized by lysosomal dysfunction 28-31. Surprisingly, although lysosomes seem to be primarily disturbed by Dox, none of the approaches to date have been targeted to this cell compartment. Thus, our strategy relying on the use of acidic nanoparticles offers a new opportunity to limit Dox-induced cardiac dysfunction, and it opens up new avenues for designing new approaches for the treatment of heart failure. However, although nanoparticles have made significant strides in cancer research 32,33, there is a scarcity of preclinical studies using nanomedicine for treating cardiovascular diseases. Indeed, most animal studies highlight the high biocompatibility and safety of silica nanoparticles, but their *in vivo* administration remains a challenge for targeting specific organs, especially the heart 20. Conventional administration routes (gavage, intravenous, intraperitoneal) lead to very low cardiac targeting, while more specific ways (intracoronary, intrapericardial, or intramyocardial) exhibit numerous shortcomings such as complex operating processes and invasiveness. To counteract the aforementioned issues, we administered nanoparticles through intratracheal nebulization, as described by Miragoli and colleagues 34. Accordingly, this technique allows for efficient cardiac targeting, as we observed a decrease of NPs-PLGA amount in lungs that paralleled cardiac accumulation. Another recent study indicated that inhalation of NPs by mice in nebulizer chambers allowed for an intrapulmonary translocation to the heart through alveolar epithelial cells and endothelial cells, suggesting that pulmonary circulation is an effective and non-invasive administration route to drive nanoparticles to the heart 35. Of note, the size of nanoparticles also influences their biodistribution and their ability to trigger an inflammatory response *in vivo*. Within this line, studies reveal that NPs with a diameter greater than 150-200 nm are quickly eliminated from the circulation and mainly accumulate in the spleen 36,37. Accordingly, our results showed there was no splenic accumulation of NPs-PLGA after administration in mice. Therefore, pulmonary-administered NPs-PLGA represent attractive therapeutic tools to prevent Dox cardiotoxicity.

Various therapies, classified as primary or secondary prevention, have been tested against Dox side effects. Primary prevention describes the strategy to act during anticancer therapy and represents an ideal strategy to avert Dox-induced cardiac damage. To date, the iron chelator and topoisomerase 2β modulator Dexrazoxane is the only approved primary cardioprotectant for preventing anthracycline-induced cardiac side effects. However, citing reports showed that the latter might increase the incidence of myelodysplastic syndrome and secondary cancers 38. Therefore, the existing treatments for Dox cardiomyopathy consist in secondary prevention to limit the progression of symptoms with commonly used neurohormonal blocking drugs 3. In this study, we demonstrate that administering acidic nanoparticles concurrently with Dox therapy resulted in reduced cardiac dysfunction and adverse remodeling. This effect was achieved by preventing crucial markers of Dox cardiotoxicity, such as cardiomyocyte atrophy, cardiomyocyte death, and cardiac fibrosis.

Importantly, the chemotherapeutic effects of Dox in three different cancer cell lines remained unaltered by NPs-PLGA, indicating that NPs-PLGA hold promise as potential primary cardioprotectants for cancer patients undergoing anthracycline treatment. The lack of impact on the anti-tumorigenic effects of Dox by NPs-PLGA could be attributed to various factors. Previous evidence showed that Dox anticancer effects and Dox cardiotoxicity occurred through distinct signaling mechanisms, although some overlap exists 39. In cancer cells, Dox apoptotic effects are mainly mediated by DNA intercalation and topoisomerase inhibition while in post-mitotic cardiomyocytes, Dox cardiotoxicity seems to rely more specifically on mitochondrial dysfunction and autophagy-lysosome dysregulation 39. This hypothesis is supported by the observation that many tumors have boosted autophagy/lysosome pathway to sustain their survival, growth and high metabolic demand. Such adaptation involves profound remodeling of the lysosomal compartment with an increased number and volume of lysosomes, an increased activity of lysosomal enzymes such as cathepsins, V-ATPase and Lamp1 40,41 and a hyperacidification of lysosomes 42. Hence, tumor cells might be more resistant to Dox-induced autophagy impairment than cardiac cells and NPs-PLGA will have no effect in such hyperacidic lysosomal environment.

Overall, our study highlights the importance of lysosomal dysfunction as a major driver of Dox cardiotoxicity. We propose cardiac nanomedicine as an original approach that may improve cardiac outcomes in cancer patients treated with anthracyclines. Finally, our data pave the way for the development of similar therapeutic strategies in the prevention of other lysosomal-related cardiac hereditary or chronic diseases.

## Materials and Methods

### Materials

Acetonitrile, 3-aminopropyltriethoxysilane (APTES), *poly(lactic-co-glycolic acid*) (PLGA-Resomer™-RG502 H), 1-éthyl-3-(3-diméthylaminopropyl)carbodiimide (EDC), dimethylformamide (DMF), and chemicals (Bafilomycin A1, doxorubicin) were purchased from Sigma Aldrich (St Quentin Fallavier, France). N-hydroxysuccinimide (NHS) was obtained from Fluka.

### Nanoparticles preparation and characterization

SiO_2_ nanoparticles (NPs) were purchased from Chromalys (Toulouse, France - ref Lumilys525). Sub-Micronic SiO_2_ particles (SMPs) were elaborated according to the protocol described by Rahman et al 43. Both types of particles were functionalized with PLGA. Briefly, particles (50 mg for NPs, 100 mg for SMPs) were suspended by sonication in acetonitrile (15 mL for NPs, 30 mL for SMPs). APTES (250 µL for NPs, 500 µL for SMPs) was added dropwise, and the suspension was agitated at 50°C for 24 h. Particles were recovered by centrifugation (4000 g for 10 min), washed three times in ethanol, and dried under vacuum. Then, these amine-modified nanoparticles (30 mg for NPs, 80 mg for SMPs) were suspended in DMF (15 mL for NPs, 80 mL for SMPs). In parallel, PLGA (73 mg for NPs, 194.6 mg for SMPs) was dissolved in DMF (15 mL for NPs, 80 mL for SMPs), and EDC (9.3mg for NPs, 24.8 mg for SMPs) and NHS (0.7 mg for NPs, 1.86 mg for SMPs) were added to the PLGA solution. Then, the PLGA solution was added to the particle suspension, which was agitated at 40°C for 2h. The particles were recovered by centrifugation (4000 g for 10 min), washed three times with distilled water, and dried under vacuum. Particle shape and size were examined via transmission electron microscopy (TEM), using a Philips Model CM20 microscope (Eindhoven, The Netherlands) or scanning electron microscopy (SEM, JEOL JSM 7800F). Size distribution was assayed by measuring the size of 100 particles using Image J software. Zeta potentials were measured in water by dynamic light scattering (DLS) using a Zetasizer Nano (Malvern Instruments, Malvern, UK). Chemical grafting of PLGA molecules on particle surfaces was analyzed by Fourier-transform infrared spectroscopy (FTIR), using a Perkin Elmer 100 Series spectrometer.

### Nanoparticles pH titration

NPs-PLGA were diluted in 20 mM pH 7.4 or pH 6.0 phosphate buffer saline (1 mg/mL), and the pH was measured at 0, 12, 24, 36 and 48h using a pH meter. All measurements were performed in triplicates.

### Cell culture and transfections

Neonatal rat ventricular myocytes (NRVMs) were obtained from neonatal rats aged 1-2 days old. The neonatal rats were euthanized, and their hearts were excised, followed by the removal of the atria. Primary culture of NRVMs was subsequently performed as previously described 44. Rat H9C2 cardiomyoblasts (American Type Culture Collection, Rockville, U.S.A.) were used as an animal-free alternative sharing many physiological properties of primary cardiac cells. Plasmid transfections with GFP-RFP-LC3, RFP-LAMP1 or pLJC5-TMEM192-3xHA constructs (Addgene) were performed using Lipofectamine 2000 reagent (Life Technologies) in Opti-MEM™ transfection medium (Gibco).

### Intracellular uptake rate (FACS)

Particles were solubilized in water and sonicated for 15 cycles of 1 min to facilitate mixing and avoid aggregation. Then, after exposure to MPs-PLGA-FITC or NPs-PLGA-FITC, cells were trypsinized, thoroughly rinsed in PBS, and analyzed with a FACSVerse flow cytometer (BD Biosciences). A minimum of 30,000 cells were analyzed after the exclusion of cellular debris. The FACSuite software was used for data acquisition.

### Lysosomal immunoisolation

The protocol for Lyso-IP was adapted from Abu-Remaileh et al 10. Briefly, 3 million NRVMs were transfected with pLJC5-TMEM192-3xHA plasmid for 24h, and incubated or not with NPs-PLGA-FITC for 24 additional hours. After washing with cold PBS + 3.6% OptiPrep (Sigma Alrich - D11556) in the presence of anti-proteases and anti-phosphatases, NRVMs were scraped in 600 µL. Then, mechanical lysis was performed with a 23G syringe, and samples were gently homogenized with 20 strokes of a Dounce homogenizer. Homogenates were centrifuged at 1000g for 10 min at 4°C, and 100 µL of supernatant (corresponding to input) were kept for Western Blot analysis. The remaining supernatant was incubated with prewashed anti-HA magnetic beads (ThermoFisher - 88836) in Optiprep 3.6% PBS supplemented with anti-protease and anti-phosphatase for 1h on a rotating wheel at 4°C. Immunoprecipitates were then gently washed three times with Optiprep 3.6% PBS supplemented with anti-protease and anti-phosphatase on a DynaMag Spin Magnet. Two additional washes were performed with PBS supplemented with anti-protease and anti-phosphatase without Optiprep. Lysosomes were then recovered by elution with 0.1 mg/mL HA peptide for Western Blot analysis (corresponding to Lysosomal fraction) and fluorescence measurements using a TECAN infinite pro-F200 (Ex/em: 490 and 530 nm) to assess the presence of NPs-PLGA-FITC. The purity of lysosomes was checked with molecular markers of various subcellular compartments (lysosomes, cytosol, mitochondria, nuclei) in whole cell lysates (input) and purified lysosomes (Lysosomal fraction).

### Lysosomal pH measurement

*Acridine Orange (AO):* Acridine Orange (Sigma Aldrich), a weak base that accumulates in acidic organelles, was used to label the lysosomes. After treatments, cells were incubated with 5 µM AO for 10 min at 37˚C and washed twice with PBS. Red fluorescence (corresponding to acidic vesicle staining) was measured using a Varioskan Flash Multimode Microplate Reader with excitation/emission at 480/630 nm. Representative pictures were taken with an epifluorescence microscope (DM600 microscope, Leica).

*Lysosensor:* Lysosomal acidification was measured in cells loaded with 2µM LysoSensor yellow/blue DND-160 (Invitrogen) for 15 min at 37˚C. The LysoSensor dye is a ratiometric probe that produces yellow fluorescence in acidic environments but changes to blue fluorescence in more neutral environments. pH calibration was performed following the protocol previously established by Diwu et al 45. Briefly, cells were incubated in MES buffer (5 mM NaCl, 115 mM KCl, 1.3 mM MgSO_4_, 25 mM MES) supplemented with 10 µM nigericin and 10 µM monensin, for 10 min, with pH adjusted within 3 to 7. The samples were read in a Varioskan Flash Multimode Microplate Reader with excitation at 360 nm. The emission 440/540 nm ratio was then calculated for each sample.

### Cathepsin D activity

Cathepsin D activity was measured as an indicator of lysosomal proteolytic activity. Enzyme activity was determined with a cathepsin D activity fluorometric assay kit according to the manufacturer's protocol (Abcam ab65302). Fluorescence was then measured with a Varioskan Flash Multimode Microplate Reader at 328/460 nm (excitation/emission).

### In vivo model

All animal procedures were performed in accordance with International Guidelines on Animal Experimentation and with the French Ministry of Agriculture license. Moreover, this investigation was conducted in compliance with the guide for Care and Use of Laboratory Animals published by Directive 2010/63/EU of the European Parliament. All mice (C57BL6) were housed in temperature-controlled cages with a 12-h light-dark cycle and given free access to water and food.

Nanoparticles were administered to mice via intratracheal nebulization following the protocol of Miragoli et al 34. Briefly, mice were anesthetized with 3% isoflurane. Then, NPs-PLGA were resuspended in 100 µL of water and intratracheally aerosolized using an IA-1C microsprayer aerosolizer and FMJ-250 High-Pressure Syringe (Shanghai Yuyan Instruments Co, Ltd).

Doxorubicin treatment consisted of 4 once-weekly injections of low-dose doxorubicin (5 mg/kg) in mice to replicate the dose found in patients as previously described 7.

### Transmission Electron Microscopy

Cardiac tissues were fixed with 4% PFA + 0.025% glutaraldehyde in Sorensen buffer (0.1 M, pH=7.4) for 1h, and with 4% PFA for one additional hour. They were then incubated O/N with a sucrose buffer at 4°C. Next, excised heart samples were embedded in optimal cutting temperature compound (OCT), and 10 µm thick sections were prepared and mounted on glass slides. These sections were then observed using fluorescence microscopy to identify areas containing NPs-PLGA-FITC. The fluorescence emitted by the nanoparticles was detected at excitation/emission wavelengths of 490 nm and 530 nm. After, samples were fixed with 1% glutaraldehyde in Sorensen buffer for 30 min and post-fixed with 1% OsO4 in 0.05 M Sorensen phosphate + 0.25 M glucose + 1% OsO4 for 30 min. After dehydration, samples were embedded in epoxy resin (Epon 812). After 48h of polymerization at 60°C, ultrathin sections (70 nm) were mounted on 100 mesh copper grids and post-stained with 3% uranyl acetate in 50% ethanol before imaging with an HT7700 Hitachi electron microscope at an accelerating voltage 80 kV.

### Echocardiography

Mice were anesthetized with 2% isoflurane and subjected to non-invasive echocardiography using a Vevo2100 Visual Sonics system. Cardiac ventricular dimensions were measured in a blinded fashion on M-MODE/2D images for the number of animals indicated.

### Biodistribution

NPs-PLGA-FITC diluted in 100 µL of water were administered to mice by intratracheal nebulization. Mice were then euthanized at different times by intraperitoneal injection of a lethal dose of pentobarbital. Organs (lungs, heart, liver, kidneys, spleen), blood, and urine were collected. Organs were homogenized in soft tissue homogenizing tubes using a Bead Ruptor 12 (Omni International, 2 cycles of 30s at 6 m/s speed). All samples were centrifuged at 1500g for 10 min at 4°C. The supernatant was collected, and 50 µL of the collected sample was read in duplicate using a TECAN Infinite Pro-F200 fluorescence microplate reader at excitation/emission wavelengths of 490 nm and 530 nm.

### Immunofluorescence and histological studies

Heart tissues were embedded in OCT (Sigma-Aldrich) using ice-cold 2-Methylbutane. Immunofluorescence experiments were performed as previously described 46. Briefly, frozen sections (6 µm) were fixed in 4% paraformaldehyde, followed by permeabilization and blocking in PBS with 0.02% FBS, 1% BSA, and 0.3% Triton X-100 at RT. Sections were immunostained O/N with the following antibodies: anti-alpha actinin (Abcam ab9465), anti-LC3 (Cell Signaling Technology #3868), anti-Vinculin (Sigma-Aldrich, V9131) followed by secondary Alexa Fluor antibodies (Molecular Probes). Nuclei were counterstained with DAPI. Images were acquired by confocal Microscope Zeiss LSM 780 and ZEN image analysis software (Zeiss) or digitized with a Hamamatsu NanoZoomer, and fluorescence intensity was quantified by ImageJ software. Average cardiomyocyte area was measured manually after vinculin staining on two different regions (150-200 cells counted per heart). To assess fibrosis, the hearts were transversely sectioned at a thickness of 6 μm. These sections were then fixed using 4% paraformaldehyde and subsequently stained with Sirius Red. Slides were scanned with Hamamatsu NanoZoomer, and fibrosis was quantified using ImageJ software (RSB). The positively stained area with Sirius Red, indicating fibrosis, was expressed as a percentage of the total area in the scanned slides. Apoptosis was evaluated using the TUNEL staining method with the In Situ Cell Death Detection Kit from Roche, following the manufacturer's instructions. The proportion of apoptosis was quantified by calculating the percentage of apoptotic nuclei relative to the total number of nuclei observed.

### Western blots

H9C2, NRVMs, or heart homogenates were lysed in RIPA buffer (50 mM Tris pH 7.2, 500 mM NaCl, 1% Triton X-100, 1 mM EDTA, protease inhibitors (Roche 11873580001), phosphatase inhibitors (Sigma-Aldrich, P0044 and P5726). Equal protein amounts were subjected to sodium dodecyl sulfate-polyacrylamide gel electrophoresis (SDS-PAGE) in 10-15% gels depending upon protein's molecular weights. After electrophoresis, proteins were transferred to nitrocellulose membranes and incubated with the following antibodies: anti-VDAC1 (#4866), anti-H3 (#9715), anti-HA (#3724) anti-GAPDH (#5174), anti-LC3B (#2775), anti-cleaved caspase 3 (#9661) and anti-p-p53 (#12571) from Cell Signaling Technology, anti-p62 (#H00008878-M01) from Abnova, anti-CTSD (#ab75852), anti-4-HNE (#ab46545) and anti-Oxphos (#ab110413) from Abcam. Proteins were detected by chemiluminescence with a Bio-Rad ChemiDoc XRS^+^ camera. Relative densities were quantified using the ImageLab 5.2.1 software (Bio-Rad). All data were normalized to internal controls.

### Autophagosomes/autolysosomes visualization

Autophagic flux was evaluated in RFP-GFP-LC3-transduced cells. Representative images were captured with an epifluorescence microscope (DM600 microscope, Leica) and quantification of yellow puncta (autophagosomes) and red puncta (autolysosomes) was performed using Image J software.

### Mitochondrial membrane potential

Mitochondrial membrane potential was evaluated using JC-1 probe (#ENZ-52304, Enzo Life Sciences). Before the end of treatments, cells were loaded with the JC-1 probe at a concentration of 5 µg/ml for 10 min at 37°C. Then, the medium was replaced by HBSS, and the fluorescence signals were recorded using a fluorimeter TECAN Infinite Pro-F200. The formation of red aggregates was measured at 535 nm (excitation wavelength) with emission at 590 nm, while the presence of green monomers was measured at 485 nm (excitation wavelength) with emission at 530 nm.

### Mitochondrial oxidative stress

Mitochondrial ROS were measured by mitoSOX probe (Invitrogen, Molecular Probes). Briefly, the cells were loaded with mitoSOX probe at a final concentration of 5 µM and incubated for 30 min after the indicated treatments. Cells were resuspended in HBSS before measuring the fluorescence using a Tecan plate reader at excitation/emission wavelengths of 400 nm and 610 nm.

### Cell death

To quantitatively assess cardiomyocyte necrosis, the release of lactate dehydrogenase (LDH) in the cell culture medium was measured using the LDH cytotoxicity assay kit from Biovision, following the manufacturer's instructions. For apoptosis assessment in NRVMs, caspase-3 activation was measured using a commercial kit from Biotium, following the manufacturer's instructions. This kit allows for the detection of caspase-3 activity as a marker of apoptosis in the cells.

### Cancer cells analysis

AY27, 4T1, and U937 cancer cell lines were plated at a density of 20,000-40,000 cells per well. After 24 h, cells were treated with Dox (5 µM) with or without NPs-PLGA and incubated for an additional 32 h in Incucyte® live-cell analysis (Sartorius). For proliferation experiments, phase pictures were taken using live-cell time-lapse imaging every 2 h. Then, cell proliferation was quantified with cell-by-cell analysis software. For apoptosis, cells were treated with Annexin V 488 Conjugate according to the manufacturer's protocol (Biotium). Fluorescence was measured with Incucyte® live-cell analysis (Sartorius) with excitation/emission at 490/515 nm.

### Statistical analysis

Data were analyzed using GraphPad Prism 9.4.1 software. Statistical analysis was carried out using T-test, 1-way or 2-way ANOVA with the Tukey post hoc test, when appropriate. The results are shown as the mean ± SEM. Values of p<0.05 were considered as significant.

## Supplementary Material

Supplementary figures.

## Abbreviations

4-HNE: 4-hydroxynonenal
APTES: (3-Aminopropyl) triethoxysilane
Baf A1: bafilomycin A1
FDA: food and drug administration
Dox: doxorubicin
EF: ejection fraction
FITC: fluoresceine isothiocyanate
FTIR: Fourier-transform infrared spectroscopy
FS: fractional shortening
GFP: green fluorescent protein
HA: hemagglutinin
LAMP1: lysosomal-associated membrane glycoprotein 1
LC3: microtubule-associated protein 1A/1B-light chain 3
NPs: nanoparticles
NRVMs: Neonatal Rat Ventricular Myocytes
OXPHOS: oxidative phosphorylation
PLGA: poly(lactic-co-glycolic acid)
PBS: phosphate buffer saline
RFP: red fluorescent protein
ROS: reactive oxygen species
SEM: scanning electron microscopy
SiO_2_: silica
SMPs: Sub-micronic particles
TEM: transmission electron microscopy
TFEB: transcription factor EB
TMEM192: transmembrane protein 192
Veh: vehicle
