# Supplementary material for: Inhalation of acidic nanoparticles prevents doxorubicin cardiotoxicity through improvement of lysosomal function
Source: Theranostics. 2023 Oct 2;13(15):5435–51. doi: 10.7150/thno.86310 (PMC10614672; doi:10.7150/thno.86310)
Supplement: Supplementary file 1 — Supplementary figures. [file thnov13p5435s1.pdf]

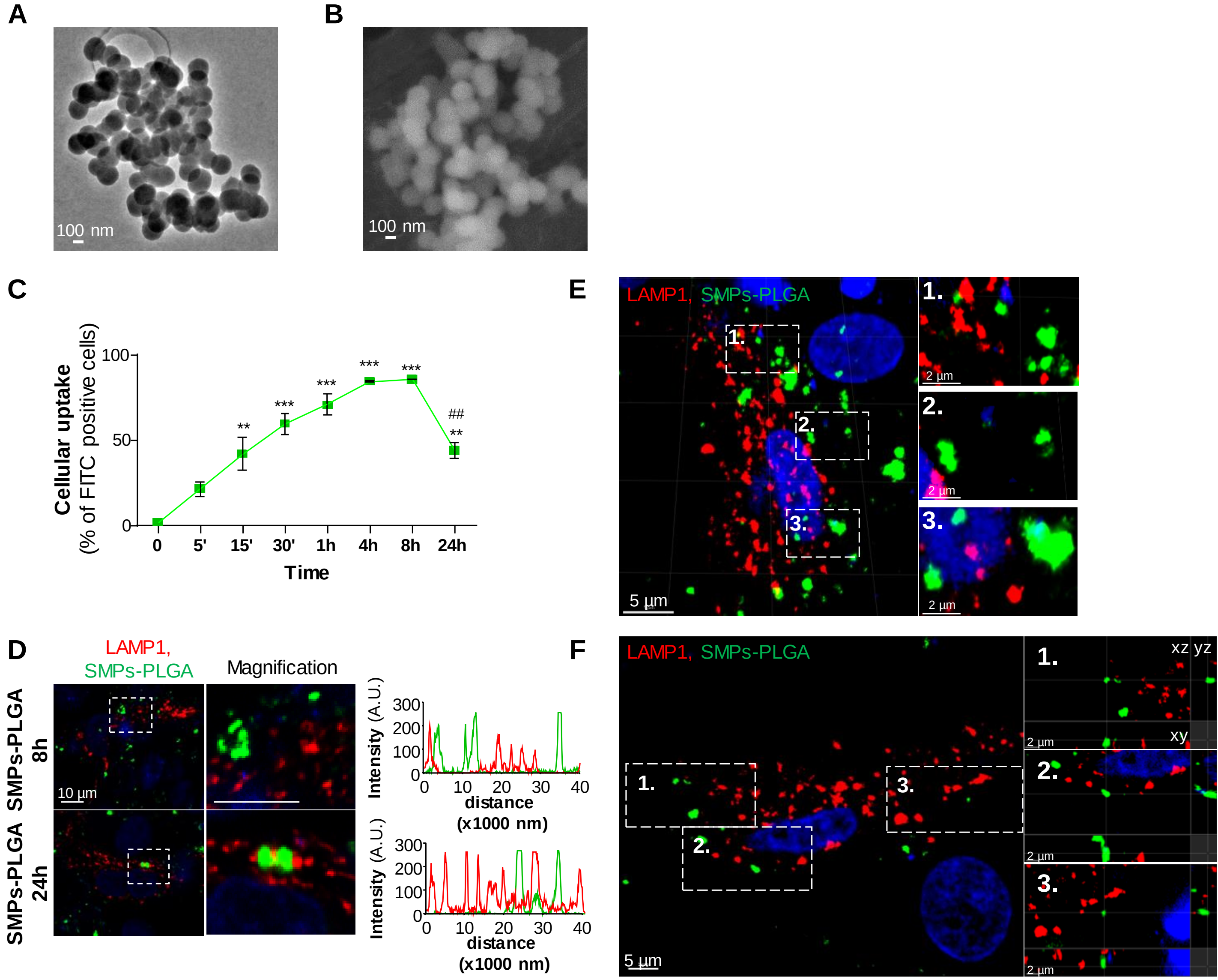

Figure S1: SMPs-PLGA exhibit extra-lysosomal localization in NRVMs

(A) Transmission electron microscopy (TEM) and **(B)** Scanning electron microscopy (SEM) of SMPs-PLGA (Scale Bar = 100 nm). **(C)** Intracellular uptake rate of SMPs-PLGA-FITC in NRVMs (n = 3). **(D)** Left panel: Representative confocal images of NRVMs transfected with LAMP1-RFP (red) and incubated with SMPs-PLGA-FITC (green) for 8 and 24 h. Hoechst 33258 (blue) was used as counterstaining. Scale Bar = 10 µm. Right panel: Intensity profile graphs of SMPs-PLGA-FITC and LAMP1-RFP. **(E)** 3-dimensional reconstruction of stack images of NRVMs transfected with LAMP1-RFP (red) and incubated with SMPs-PLGA-FITC (green) for 24 h. Hoechst 33258 (blue) was used as counterstaining. Scale Bar = 5 µm for whole images, 2 µm for magnified areas. **(F)** Orthogonal projection of stack images of NRVMs transfected with LAMP1-RFP (red) and incubated with SMPs-PLGA-FITC (green) for 24 h. Hoechst 33258 (blue) was used as counterstaining. Scale Bar = 5 µm for whole images, 2 µm for magnified areas. Data are expressed as means ± SEM (**p < 0.01, ***p < 0.001 vs t = 0; ##p < 0.01, vs t = 8 h)

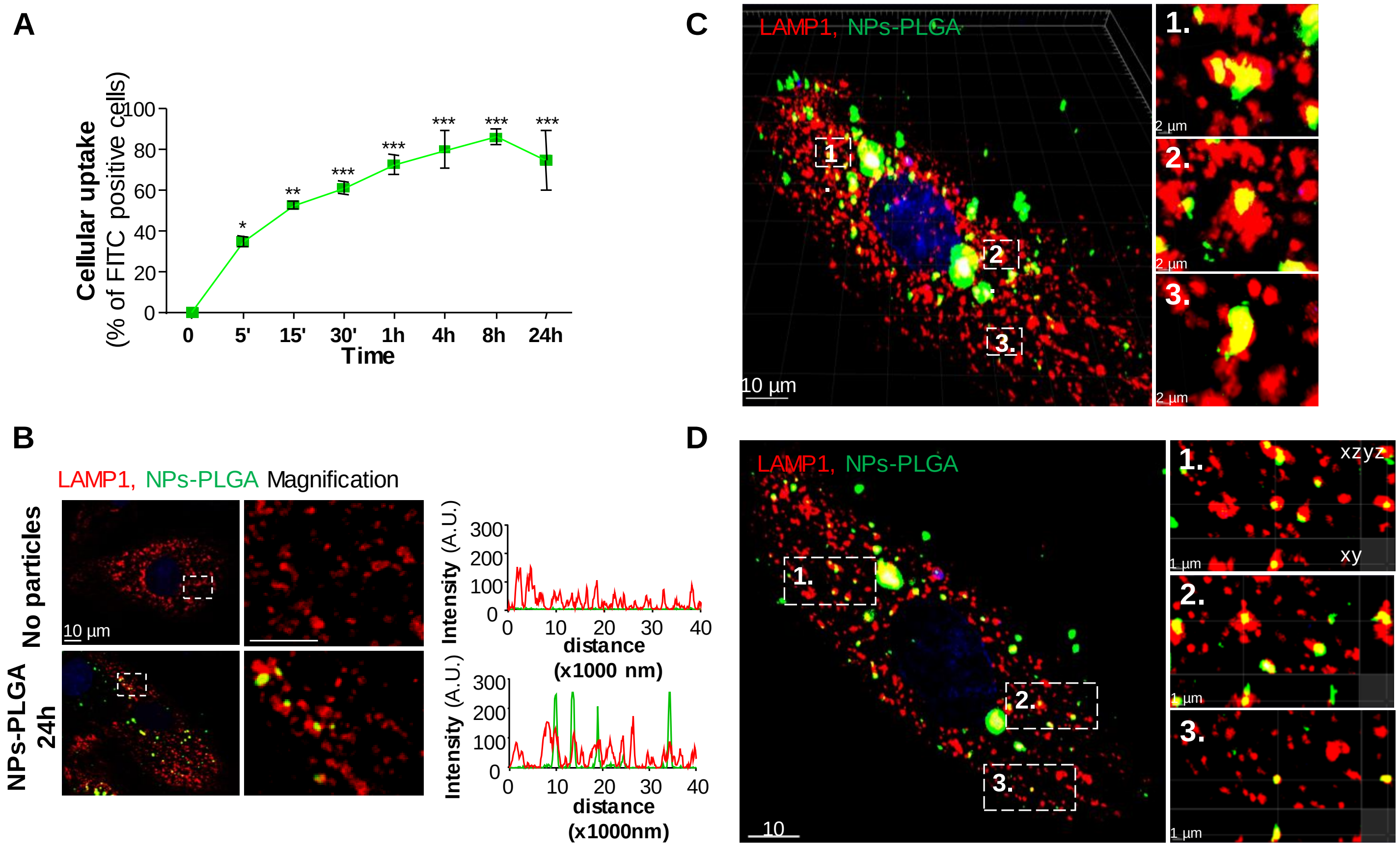

Figure S2: NPs-PLGA address lysosomal compartment in H9C2

(A) Intracellular uptake rate of NPs-PLGA-FITC in H9C2 (n = 3). **(B)** Left panel: Representative confocal images of H9C2 transfected with LAMP1-RFP (red) and incubated or not with NPs-PLGA-FITC (green) for 24 h. Hoechst 33258 (blue) was used as counterstaining. Scale Bar = 10 µm. Right panel: Intensity profile graphs of NPs-PLGA-FITC colocalization with LAMP1-RFP. **(C)** 3-dimensional reconstruction of stack images of H9C2 transfected with LAMP1-RFP (red) and incubated with NPs-PLGA-FITC (green) for 24 h. Hoechst 33258 (blue) was used as counterstaining. Scale Bar = 10 µm for whole images, 2 µm for magnified areas. **(D)** Orthogonal projection of stack images of H9C2 transfected with LAMP1-RFP (red) and incubated with NPs-PLGA-FITC (green) for 24 h. Hoechst 33258 (blue) was used as counterstaining. Scale Bar = 10 µm for whole images, 1 µm for magnified areas. Data are expressed as means ± SEM (*p < 0.05**p < 0.01, ***p < 0.001 vs t = 0)

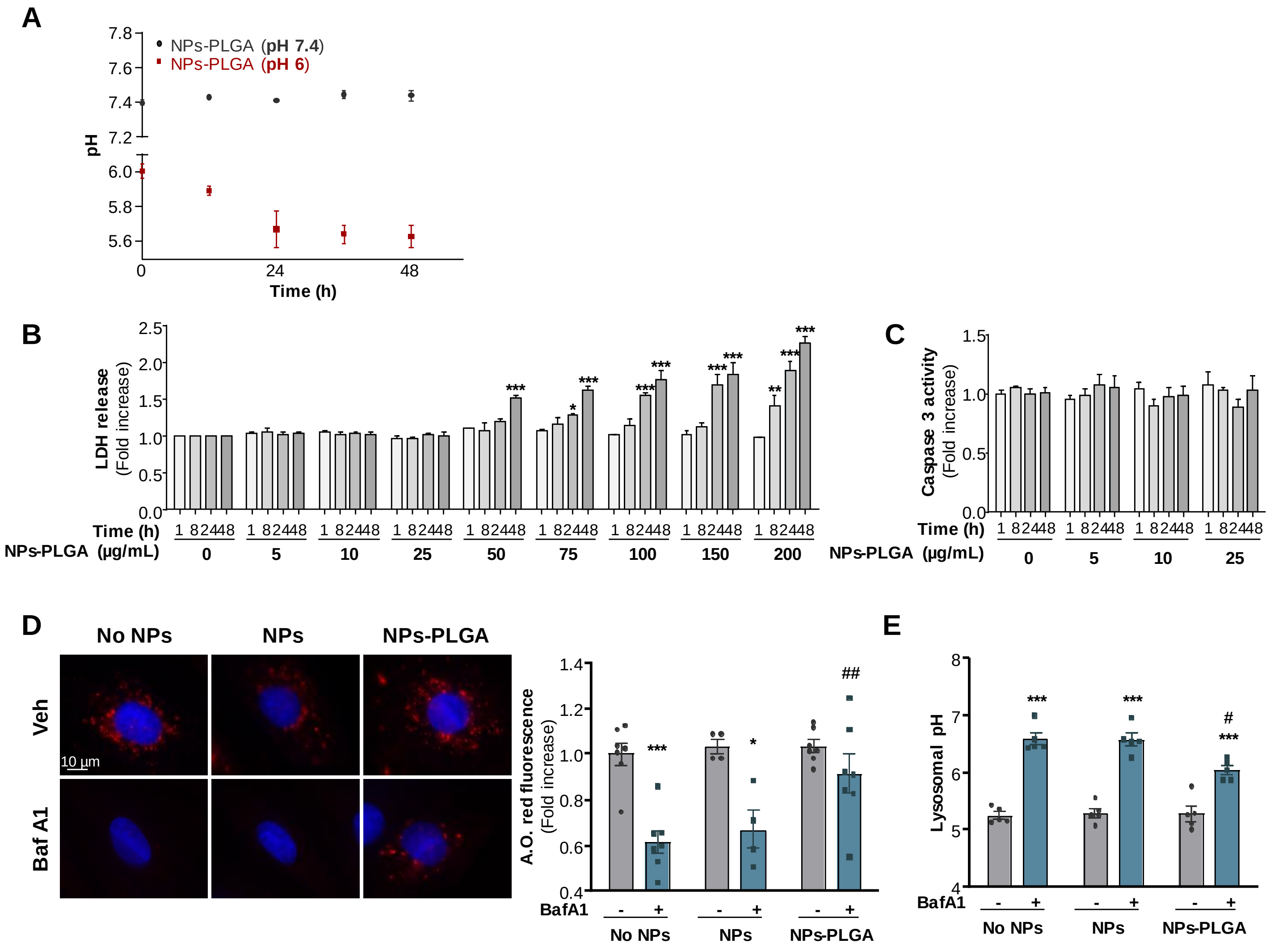

Figure S3: NPs-PLGA prevent BafA1-induced lysosomal dysfunction in H9C2

(A) pH changes of NPs-PLGA in 20 mM pH 6 or 7.4 PBS solution over a 48h period (n = 3) **(B)** LDH release and **(C)** Caspase 3 activity in H9C2 incubated with increasing times and concentrations of NPs-PLGA (n = 4). **(D-E)** H9C2 were pre-incubated O/N with 25 µg/mL of NPs or NPs-PLGA and treated with Bafilomycin A1 (100 nM, 1 h). **(D)** Left panel: Representative images of Acridine Orange red fluorescence. Scale Bar = 10 µm. Right panel: Fluorimeter measurements of Acridine Orange red staining (n = 4-7). **(E)** Fluorimeter measurements of Lysosensor yellow/blue staining (n = 5). Excitation was measured at 360 nm and the ratio of emission 440/540 nm was calculated. Data are expressed as means ± SEM (*p < 0.05, **p < 0.01, ***p < 0.001 vs Veh or t = 0; #p < 0.05, ##p < 0.01 vs BafA1)

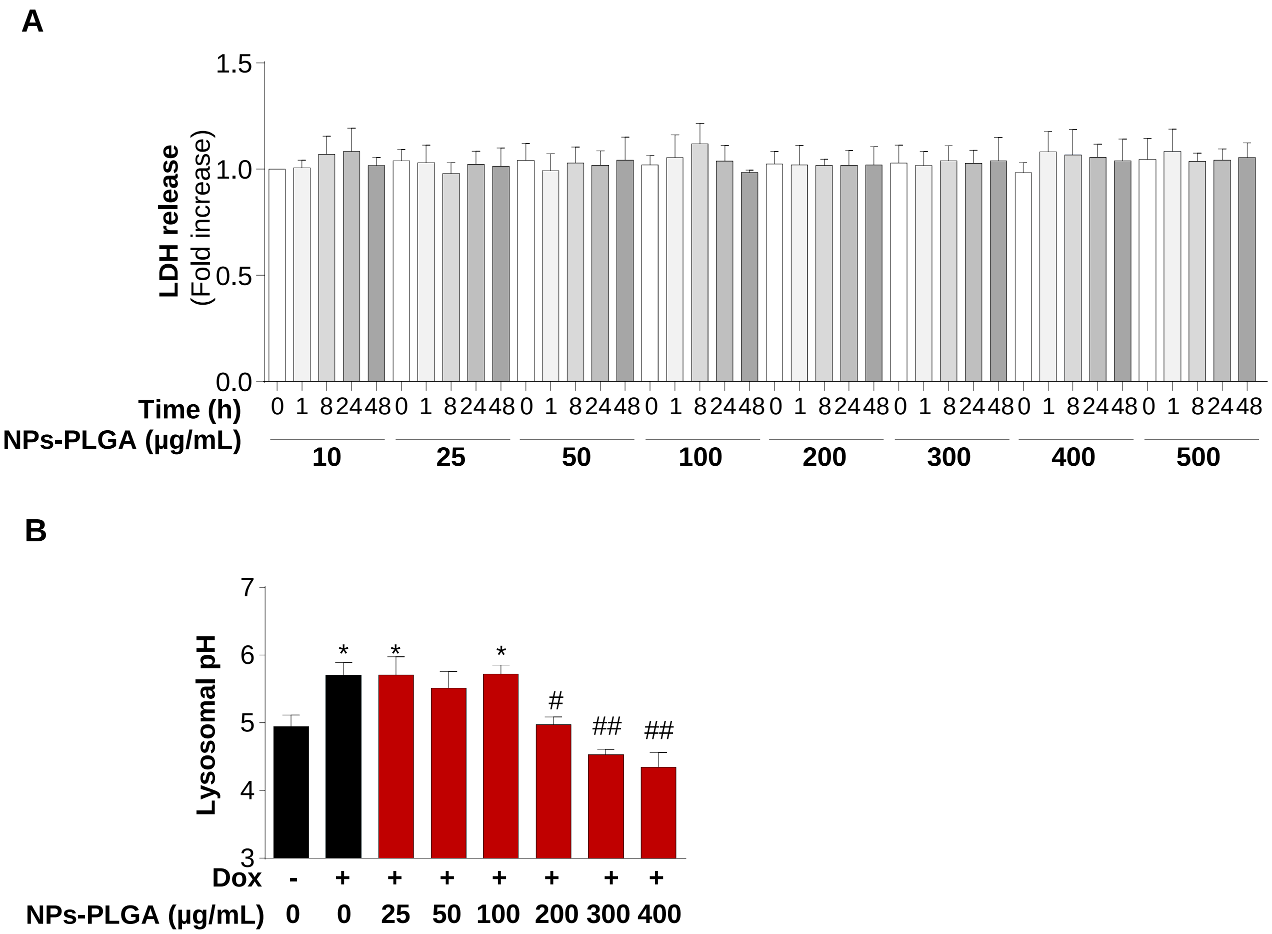

Figure S4: NPs-PLGA are not cytotoxic in NRVMs

(A) LDH release in NRVMs incubated with increasing times and concentrations of NPs-PLGA (n = 4). **(B)** Fluorimeter measurements of Lysosensor yellow/blue staining in NRVMs incubated O/N with increasing doses of NPs-PLGA in the presence of Dox (1 µM, 10 h) (n = 8). Excitation was measured at 360 nm and the ratio of emission 440/540 nm was calculated. Data are expressed as means ± SEM (*p < 0.05 vs Veh; #p < 0.05, ##p < 0.01 vs Dox)

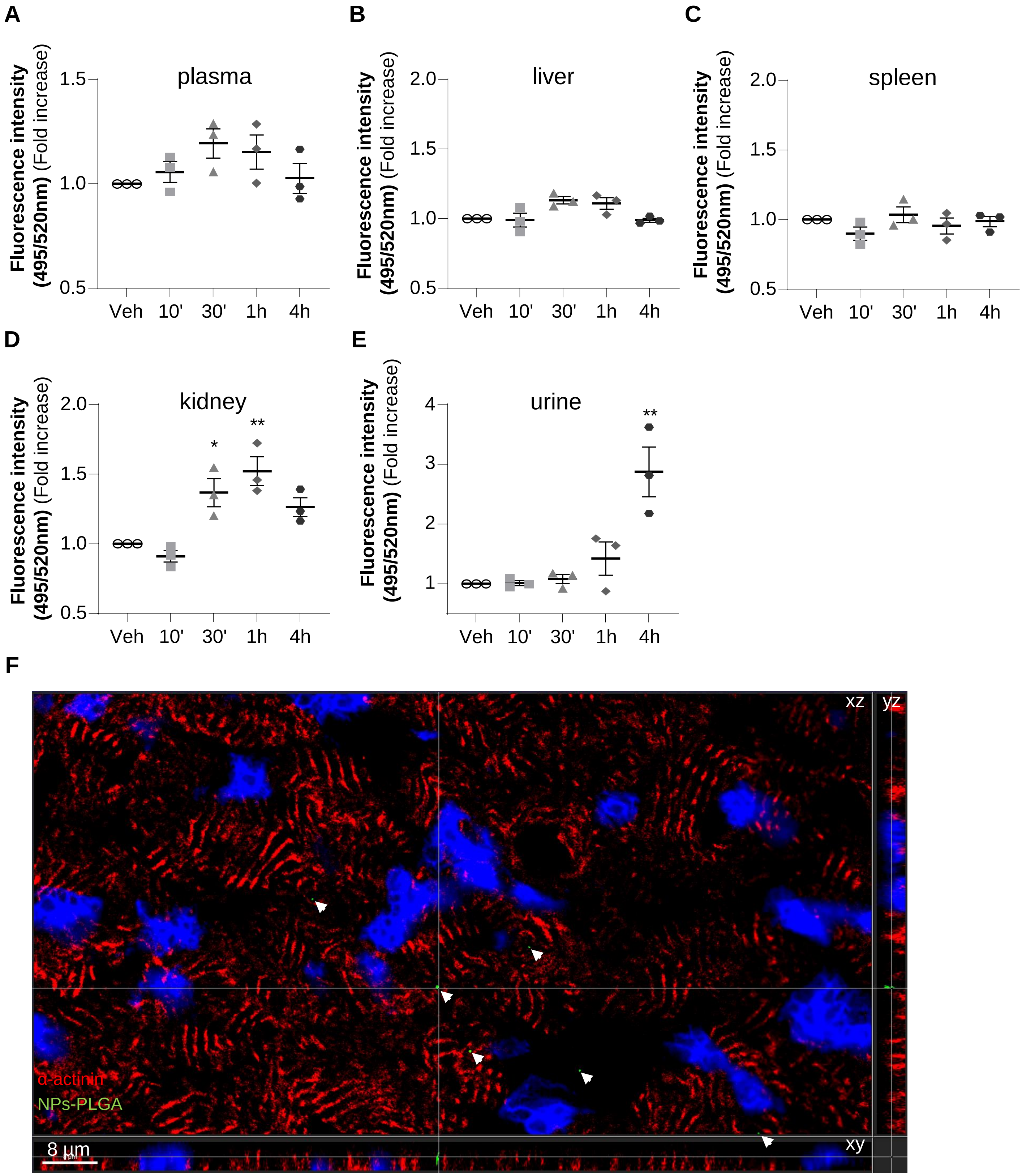

Figure S5: Biodistribution of NPs-PLGA after intratracheal nebulization in mice

(A-E) Time-course quantification of green fluorescence in different organs of mice treated with 1 mg/kg of NPs-PLGA-FITC via intratracheal nebulization (n = 3) with **(A)** plasma, **(B)** liver, **(C)** spleen, **(D)** kidney, and **(E)** urine. Data are normalized per mg of tissue or per volume. **(F)** Orthogonal projection of stack images of heart tissues from mice collected 1 h after intratracheal nebulization of Veh or 1 mg/kg of NPs-PLGA-FITC. Green: NPs-PLGA-FITC, red: alpha-actinin, blue: DAPI. Scale bar = 8 µm. Data are expressed as means ± SEM (*$p < 0.05$, **$p < 0.01$ vs Veh)

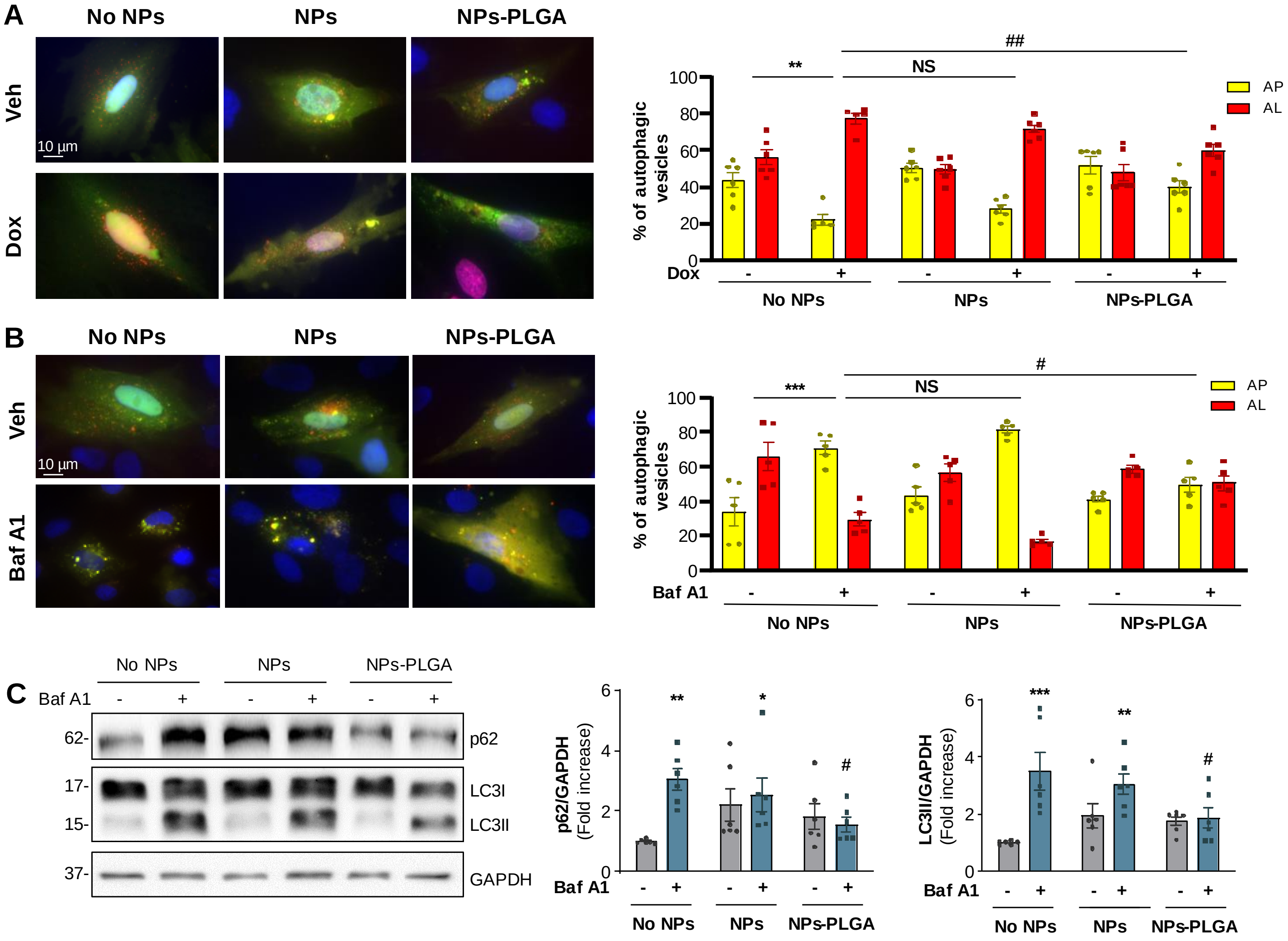

Figure S6: NPs-PLGA prevent Dox and BafA1-induced autophagic flux blockade in H9C2

(A-C) H9C2 were pre-incubated O/N with Veh or 25 µg/mL of NPs or NPs-PLGA and treated or not with **(A)** Doxorubicin (1 µM, 16 h) or **(B-C)** Bafilomycin A1 (100 nM, 1 h). **(A)** Left panel: Double immunofluorescence imaging of RFP-GFP-LC3-transfected H9C2. Right panel: Quantification of yellow puncta (AP, autophagosomes) and red puncta (AL, autolysosomes) for each condition (n = 5-6). **(B)** Left panel: Double immunofluorescence imaging of RFP-GFP-LC3-transfected H9C2. Right panel: Quantification of yellow puncta (AP, autophagosomes) and red puncta (AL, autolysosomes) for each condition (n = 5). **(C)** Left panel: Representative immunoblots and right panel: quantifications of p62 and LC3-II expression in H9C2 (n = 6). GAPDH was used as a loading control. Data are expressed as means ± SEM (*p < 0.05, **p < 0.01, ***p < 0.001 vs Veh; #p < 0.05, ##p < 0.01 vs Dox or BafA1)

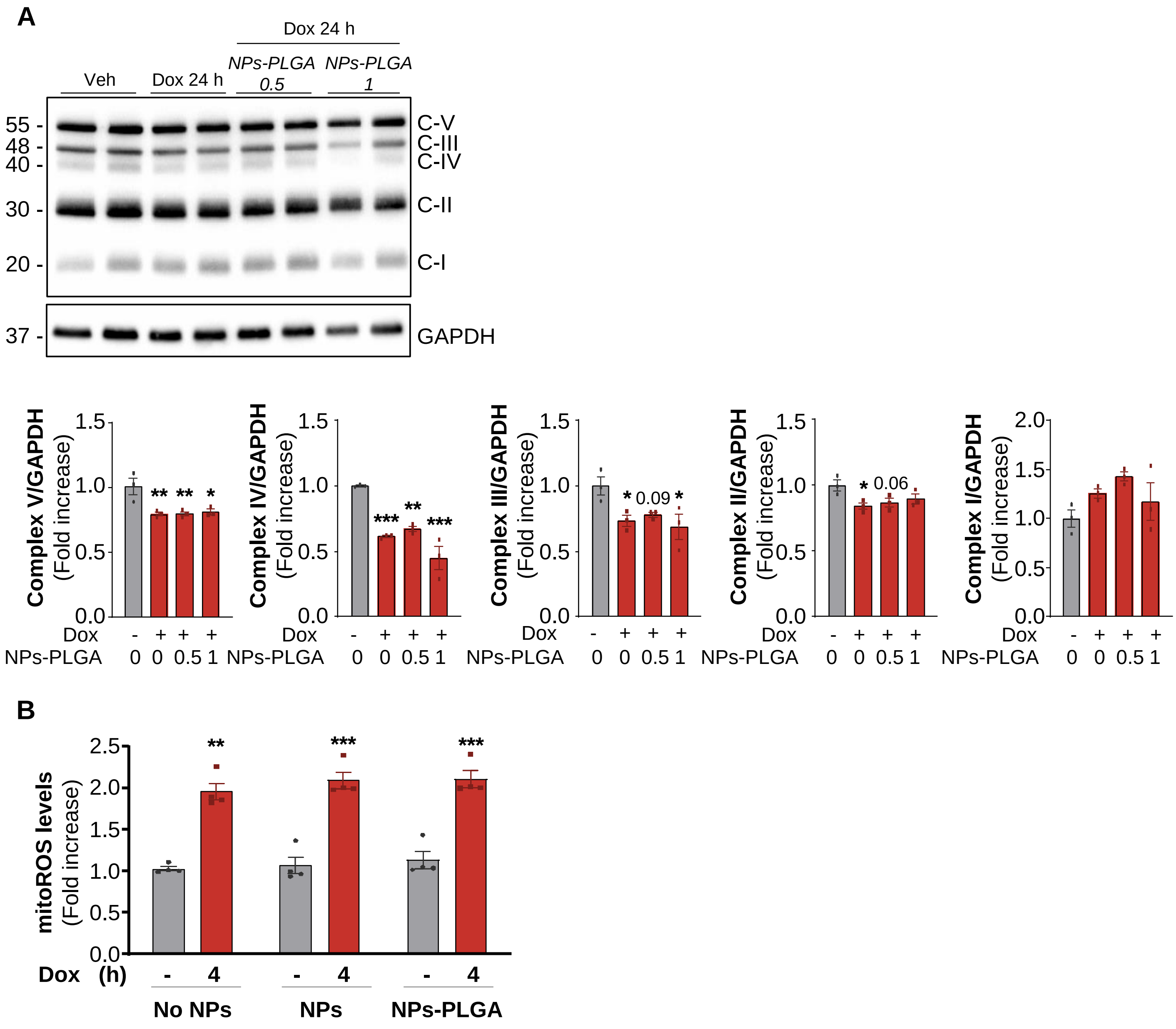

Figure S7: NPs-PLGA do not prevent Dox-induced early mitochondrial dysfunction

(A) Left panel: Representative immunoblots and right panel: quantifications of OXPHOS complexes expression in cardiac homogenates of mice 24 h after Veh or Dox (5 mg/kg) ± NPs-PLGA (0.5 or 1 mg/kg) administration (n = 3). Dox and NPs-PLGA were administered at the same time. GAPDH was used as a loading control. **(B)** Quantification of mitoROS assessed by fluorimeter measurements of mitoSOX fluorescence on H9C2 treated with Veh or Dox (1 µM, 4 h) (n = 4). Data are expressed as means ± SEM (*$p < 0.05$, **$p < 0.01$, ***$p < 0.001$ vs Veh)

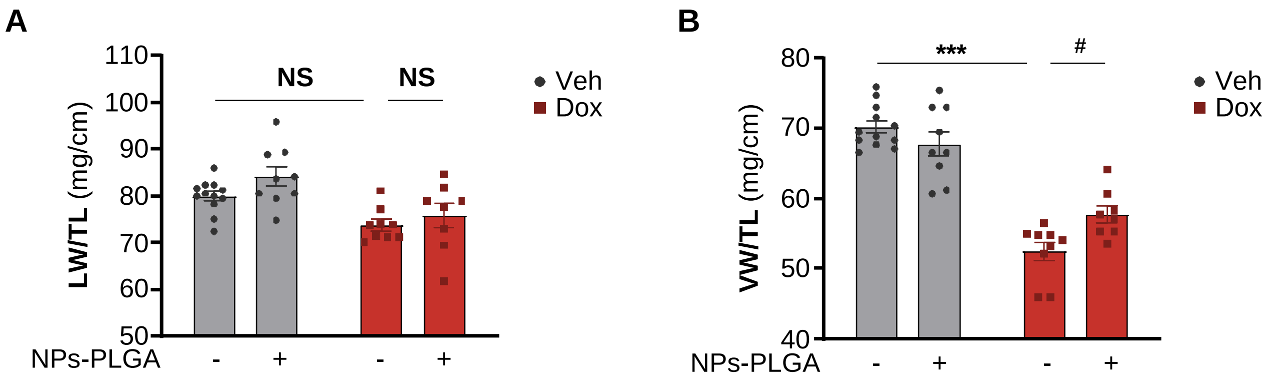

Figure S8: Morphometric parameters of Dox-treated mice after IT nebulization of NPs-PLGA

(A-B) Mice were treated with 4 once-per-week injection of Veh or Dox (5 mg/kg) ± NPs-PLGA (1 mg/kg). Dox and NPs-PLGA were administered at the same time. Morphometric parameters with **(A)** Lung weight-to-Tibia length ratio (n = 8-12) and **(B)** Ventricles weight-to-Tibia length ratio (n = 8-12). Data are expressed as means ± SEM (***p < 0.001 vs Veh; #p < 0.05 vs Dox)
